# Hemodialysis exacerbates proteolytic imbalance and pro-fibrotic platelet dysfunction

**DOI:** 10.1038/s41598-021-91416-8

**Published:** 2021-06-03

**Authors:** Aaron J. Velasquez-Mao, Mark A. Velasquez, Zhengxiong Hui, Denise Armas-Ayon, Jingshen Wang, Moriel H. Vandsburger

**Affiliations:** 1 UC Berkeley-UCSF Graduate Program in Bioengineering, Berkeley, CA USA; 2 Department of Bioengineering, UC Berkeley, 281 Hearst Memorial Mining Building, Berkeley, CA 94721 USA; 3 Department of Biostatistics, UC Berkeley, Berkeley, CA USA

**Keywords:** Haemodialysis, End-stage renal disease

## Abstract

Multi-organ fibrosis among end stage renal disease (ESRD) patients cannot be explained by uremia alone. Despite mitigation of thrombosis during hemodialysis (HD), subsequent platelet dysfunction and tissue dysregulation are less understood. We comprehensively profiled plasma and platelets from ESRD patients before and after HD to examine HD-modulation of platelets beyond thrombotic activation. Basal plasma levels of proteolytic regulators and fibrotic factors were elevated in ESRD patients compared to healthy controls, with isoform-specific changes during HD. Platelet lysate (PL) RNA transcripts for growth and coagulative factors were elevated post-HD, with upregulation correlated to HD vintage. Platelet secretome correlations to plasma factors reveal acutely induced pro-fibrotic platelet phenotypes in ESRD patients during HD characterized by preferentially enhanced proteolytic enzyme translation and secretion, platelet contribution to inflammatory response, and increasing platelet dysfunction with blood flow rate (BFR) and Vintage. Compensatory mechanisms of increased platelet growth factor synthesis with acute plasma matrix metalloproteinase (MMP) and tissue inhibitor of MMPs (TIMP) increases show short-term mode-switching between dialysis sessions leading to long-term pro-fibrotic bias. Chronic pro-fibrotic adaptation of platelet synthesis were observed through changes in differential secretory kinetics of heterogenous granule subtypes. We conclude that chronic and acute platelet responses to HD contribute to a pro-fibrotic milieu in ESRD.

## Introduction

Chronic kidney disease (CKD) is characterized by progressive loss of kidney function culminating in end stage renal disease (ESRD). Although hemodialysis (HD) is the prevailing renal replacement therapy, it is an independent risk factor for cardiovascular disease (CVD) mortality^1–6^ beyond the traditional risk factors of diabetes, hypertension, lipoprotein levels, and smoking^7^. Potential causes of CVD-related mortality in patients with ESRD include coronary artery disease, vascular calcification with sustained local and systemic inflammatory and pro-osteogenic upregulation^5^, and uremic cardiomyopathy characterized by left ventricular hypertrophy, focal scarring, and diffuse interstitial fibrosis^8–11^. Further, mitochondrial disease^12, 13^ secondary to CKD^14, 15^ is linked to endothelial dysfunction and extracellular matrix (ECM) remodeling. Recent studies have revealed diffuse myocardial fibrosis in response to initiation of HD^16^. Non-invasive imaging studies demonstrate correlations between cardiac fibrosis and imbalanced plasma proteolytic regulators, particularly tissue inhibitors of matrix metalloproteinases (TIMPs) TIMP1 and TIMP2, in ESRD patients^17^. In parallel, plasma biomarkers for endothelial dysfunction, oxidative stress, and inflammation^3, 18, 19^ indicate CKD progression and cardiovascular decline. Uremic retention compounds inadequately removed during HD have been identified as potential toxins driving the aforementioned pathological processes^6, 20–22^. However, cardiotoxicity from circulatory waste accumulation alone insufficiently explains the scope of adverse cardiovascular events and multi-organ fibrosis observed in HD patients, considering lower incidence in peritoneal dialysis patients^23^ despite similar residual toxin retention.

Platelets derive secretomes from megakaryocyte upregulation prior to fragmentation and shift patterns of synthesis and secretion in response to activation^24^. Prior to fragmentation, megakaryocytes differentially sort mRNA into platelets with selective emphasis on MMP and TIMP transcripts^25^. HD aberrantly and repetitively stimulates platelets^26^, however prior studies of platelets during HD and anti-platelet therapy trials focus on acute thrombus formation^27^. While thrombogenic platelet responses in HD are mitigated with anti-thrombotic agents, concomitant modulations of platelet secretion of factors implicated in tissue regeneration, fibrotic diseases, and cardiovascular stiffening^28^ remain poorly understood. Additionally, megakaryocyte-driven fibrosis may be stimulated by HD-induced release of compounds known to be inadequately removed during HD^29^. The combination of acute changes in platelet physiology and chronic adaption of platelet formation may serve as a potential mechanism underlying the development of multi-organ fibrosis and endothelial dysfunction in ESRD patients following initiation of HD.

In these experiments, we recruited ESRD patients on routine hemodialysis from DaVita clinics in the Oakland, CA area. Blood samples were obtained immediately before and after HD. Healthy controls were recruited for comparison. We quantified differences in circulating concentrations of proteolytic regulators, growth factors (GF), clotting factors, cardiovascular indicators, and inflammatory indicators in healthy versus HD patient blood and further measured changes in HD patients pre- versus post-treatment. Because of differences observed in MMPs and TIMPs between healthy and HD patient blood considering familiar differences observed in GF, cardiovascular, and inflammatory levels, we characterized platelet secretomes to determine contributions from HD. Our findings suggest acute and sustained adaptations in platelet protein synthesis and secretion from HD treatment leading to progressively pro-fibrotic behaviors. This shift in platelet phenotype may contribute to enhanced tissue remodeling in HD patients beyond conventional processes of hypertrophy, leading to heightened frequency of adverse cardiovascular outcomes.

## Results

### Hemodialysis patient plasma is proteolytically dysregulated and pro-fibrotic

**Figure 1:**
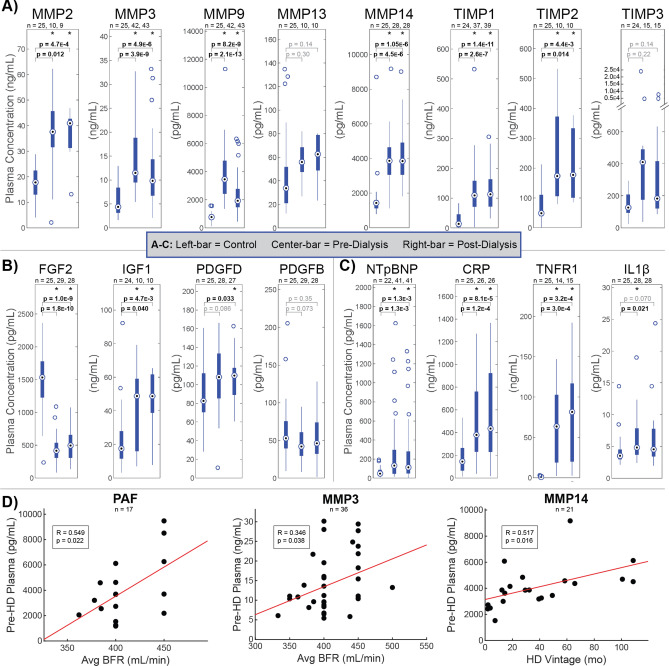
Hemodialysis patient plasma is characterized by proteolytic dysregulation and pro-fibrotic indicators. Comparison of plasma concentrations of (**A**) MMPs and TIMPs, (**B**) growth factors, and (**C**) inflammatory and cardiovascular markers between healthy control and pre-dialysis or post-dialysis samples as measured by ELISA and compared by t test. *p < 0.05. Box and whisker plots represent quartiles. (**A**) Concentrations of four MMPs (MMP2, MMP3, MMP9, MMP14) and two TIMPs (TIMP1, TIMP2) were significantly elevated in dialysis patients both before and after HD compared to healthy controls. (**B**) Significantly depressed FGF2 and significantly elevated IGF1 in dialysis patients indicate a pro-fibrotic circulatory phenotype. (**C**) Natriuretic peptides and inflammatory markers are elevated in dialysis patients as established indicators of adverse cardiovascular outcomes. (**D**) Significant Pearson correlations (*p* < 0.05) between pre-dialysis plasma concentrations and treatment parameters blood flow rate (BFR) and dialysis vintage. Positive relationships against PAF and MMPs support worsening proteolytic and coagulative dysfunctions with BFR and Vintage. Figure was prepared in Matlab R2019 (Mathworks, Nattick MA, www.mathworks.com).

HD patient plasma indicated broadly elevated proteolytic activity and regulation, a pro-fibrotic GF profile, cardiac stress, and elevated systemic inflammatory response compared to healthy plasma (Fig. 1). Concentrations of four matrix metalloproteinases (MMPs: MMP2, MMP3, MMP9, MMP14) and two tissue inhibitors of MMPs (TIMPs: TIMP1, TIMP2) were significantly elevated in dialysis patients both before and after HD compared to control values (Fig. 1A). In contrast, plasma levels of growth factors were more nuanced (Fig. 1B), with fibroblast growth factor two (FGF2) levels significantly below and insulin like growth factor 1 (IGF1) concentrations significantly above control levels. Platelet derived growth factor (PDGF) D concentrations pre-HD trended higher than control levels and were significantly elevated in post-HD plasma, while neither pre- nor post-HD PDGFB levels significantly differed from controls. In agreement with prior studies^30–33^, inflammation and cardiovascular stress measured uniformly higher in dialysis patients, shown by N-Terminal Prohormone of Brain Natriuretic Peptide (NTpBNP), C-reactive protein (CRP), tumor necrosis factor receptor 1 (TNFR1), and interleukin 1 beta (IL1β) as shown in Fig. 1C. Broad elevation of MMPs and TIMPs support enhanced tissue remodeling as an adaptive response to HD. Further, the widely sustained elevation of plasma MMPs and TIMPs post-treatment suggests a pathogenesis that is insufficiently cleared and potentially exacerbated by HD. Given prior findings that FGF2 reduces structural damage in CKD^34^, while elevated IGF1 correlates to systemic sclerosis^35^, these results suggest a pro-fibrotic phenotype in HD patient plasma. Further, given the key role PDGFs play in the genesis of vascular lesions, elevated chronic PDGFD and minor PDGF increases post-HD may contribute to a homeostatic shift toward tissue stiffening in HD patients^36^. This link between proteolytic dysregulation and fibrosis in HD patients is further supported by the mechanistic induction of TIMPs and attenuation of MMPs by PDGFs^37^. No differences were observed between dialysis patient and control plasma for the TGFβ precursor latency associated peptide (LAP), pro-platelet basic protein (PPBP)/ Beta-Thromboglobulin (βTG), Platelet-Activating Factor (PAF), Von Willebrand Factor (vWF), platelet factor 4 (PF4), Glyceraldehyde 3-Phosphate Dehydrogenase (GAPDH), Thrombopoietin (TPO), thromboxane metabolite (TXM) 11TXB2, and Interferon-γ (IFNγ). For vasodilator Prostaglandin I2 (Prostacyclin; PGI2) no difference was observed between pre-HD and healthy control levels, though post-HD levels were significantly suppressed (Fig. SF1).

### Intra-dialysis changes reveal reduced MMP and heightened TIMP1 plasma levels

**Figure 2:**
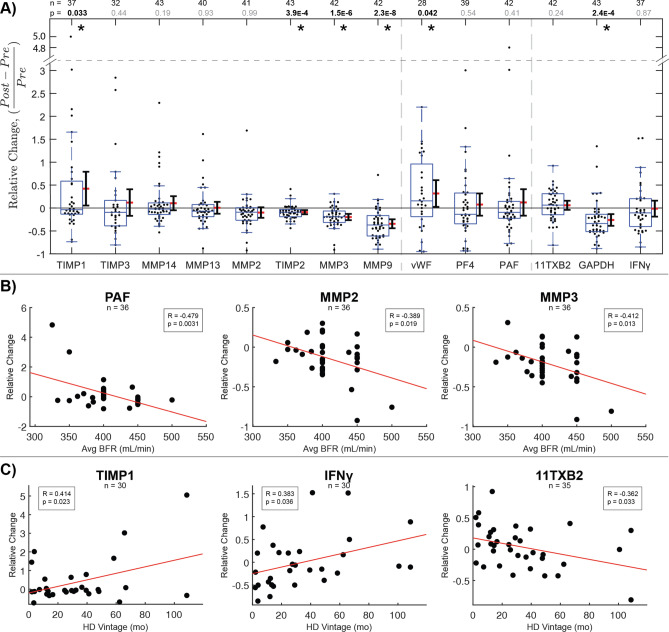
Intra-dialysis changes reveal MMP clearance and acute TIMP1 elevation that increases in severity with dialysis vintage. (**A**) Relative changes in paired plasma samples from dialysis patients pre- and post-treatment as measured by ELISA. Significant intra-dialysis increases were observed in proteolytic regulator TIMP1 and clotting factor vWF. Significant decreases were observed in TIMP2, proteases MMP3 and MMP9, and oxidative stress indicator GAPDH. Statistical significance was assessed using one-sample t test. *p < 0.05. Box and whisker plots represent quartiles. Adjacent ranges represent mean (red) ± 95% confidence interval. (**B** and **C**) Significant Pearson correlations (*p* < 0.05) between intra-dialysis plasma protein changes and BFR or Vintage, respectively. Negative relationships against PAF and MMPs show clearance with increasing BFR. Positive relationships against TIMP1 and cytokine IFNγ support increasing proteolytic inhibition and immune reactivity with Vintage, while decreasing thromboxane metabolite with Vintage reflects progression of coagulative dysfunction. Figure was prepared in Matlab R2019 (Mathworks, Nattick MA, www.mathworks.com).

The impacts of single HD sessions on circulatory biomarker accumulation were assessed by relative changes in paired plasma samples from dialysis patients pre- and post-treatment. One-sample t tests indicated plasma protein concentrations likely to change iatrogenically on average. Post-HD plasma indicated lower proteolytic activity, higher proteolytic inhibition, higher clot sensitivity, and lower glycolytic enzyme levels compared to paired pre-HD samples. Significant intra-dialytic changes were found in 2 MMPs (MMP3, MMP9), 2 TIMPs (TIMP1, TIMP2), no GFs, 1 clotting factor (vWF), 1 cardiovascular indicator (GAPDH), and no inflammatory indicators (Fig. 2). All measured proteases and proteolytic regulators decreased in a majority of HD patients, reflecting typical protein removal effects by mechanisms like membrane adsorption. However, TIMP1 significantly increased on average. Considering TIMP2, MMP3, and MMP9 significantly decrease on average, this isolates TIMP1 as a molecule that is acutely secreted into plasma during dialysis. In particular, an increased ratio of TIMP1 to MMP9 post-dialysis may link HD to increased tissue fibrosis and systemic stiffening characteristic of cardiorenal syndrome. In contrast, relative changes among measured growth factors were not statistically significant. FGF2, PDGFB, IGF1, and Connective Tissue Growth Factor (CTGF) increased in a majority of patients during HD sessions, while epidermal growth factor (EGF), LAP, PDGFD, and PPBP decreased in a majority of sessions (Fig. SF2). Among measured clotting factors, vWF levels significantly increased on average. Among cardiovascular and inflammatory indicators, GAPDH significantly decreased on average. In parallel, 11TXB2 and CRP increased in a majority of sessions, while Thrombopoietin (TPO), NTpBNP, PGI2, TNFR1, TNFα, IFNγ, and IL1β decreased in a majority of sessions, with no significant paired average changes (Fig. SF2).

### Plasma proteolytic imbalance worsens with dialysis vintage and faster blood flow rate

Pre-treatment levels of all proteins measured were compared simultaneously by multivariate linear regression (MLR) against patient age, HD Vintage defined as the number of months of regular HD treatment prior to collection, average HD blood flow rate (BFR), sex, and weight to determine potential targets and patient predictors of cyclical dialytic stress (Table ST6). Plasma levels of PAF correlated uniquely to BFR against all patient predictors (*p* = 0.017) (Fig. 1D), elevations of which may contribute to the development of microvascular dysfunction^38^ and amplification of leukocyte-induced microvascular alterations through enhanced platelet recruitment^39^. Separately, MMP3 correlated uniquely to BFR (*p* = 0.025) and MMP14 correlated uniquely to HD Vintage (*p* = 0.038) against all patient predictors, indicating increasingly disturbed proteolytic balance.

Relative plasma changes intra-HD compared to treatment parameters indicated effects and predictors of protein removal and accumulation (Table ST6). Among simultaneously compared patient predictors, BFR correlated negatively against ΔPAF (*p* = 0.018) and ΔMMP2 (*p* = 0.058) (Fig. 2B), while ΔMMP3 was predicted by both BFR (*p* = 0.020) and weight (*p* = 0.046) (Fig. SF3), reflecting acute effects of increasing prescribed HD speed. HD Vintage uniquely predicted positive ΔIFNγ (*p* = 0.043), Δ11TXB2 (*p* = 0.032), and ΔTIMP1 (*p* = 0.027), reflecting both acute changes during dialysis and longer-term chronic adaptations to repeated dialysis exposure (Fig. 2C). Positive correlations between BFR and pre-treatment PAF and MMPs suggest increasing BFR may aid short-term clearance while also contributing to long-term dysfunction. However, increasing IFNγ and decreasing 11TXB2 with vintage imply chronically enhanced inflammatory sensitivity and platelet dysfunction in response to prolonged HD treatment. The combination of chronically elevated plasma TIMP1 levels in HD patients (Fig. 1A), further acute elevation in response to dialysis (Fig. 2A), and positive correlation to dialysis vintage suggests both acute and chronic adaptations that enhance MMP inhibition and fibrotic ECM deposition^40^.

### Intra-dialysis platelet transcriptome changes reflect long-term drivers of dysfunction

**Figure 3:**
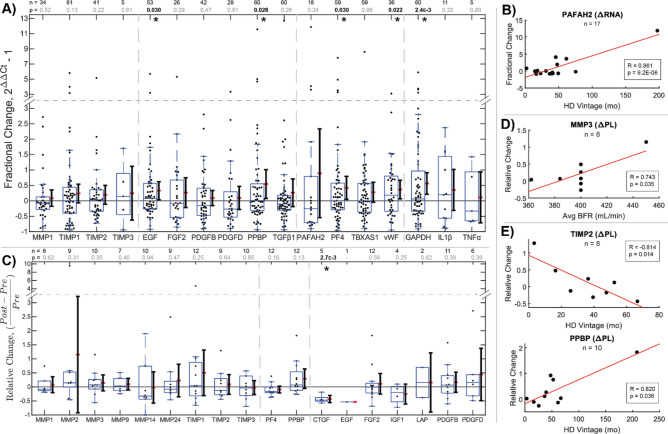
Intra-dialysis platelet transcriptome and secretome changes reflect long-term drivers of dysfunction. (**A**) Relative changes in paired platelet lysate RNA composition in dialysis patients pre- and post-treatment as measured by qPCR. Disproportionate increases in transcripts post-dialysis show selective upregulation of TIMPs and MMP1 degradation in a majority of patients. Fibro-protective EGF, pro-fibrotic PPBP, clotting factors PF4 and vWF, and GAPDH significantly increased by t test, reflecting changes affecting a diverse set of mechanisms. *p < 0.05. Box and whisker plots represent quartiles. Adjacent ranges represent mean (red) ± 95% confidence interval. (**B**) A significant Pearson correlation (*p* < 0.05) shows increasing capacity for PAF production with Vintage. (**C**) Relative changes in paired platelet lysate protein composition in pre- and post-treatment as measured by Western Blot. Statistical significance was assessed by one-sample t test. *p < 0.05. (**D** and **E**) Significant Pearson correlations (*p* < 0.05) between intra-dialysis platelet lysate secretome changes and BFR or Vintage, respectively. The positive relationship between MMP3 and BFR supports acute proteolytic enhancement in platelets, while reduction in MMP1 and TIMP2 with Vintage supports sensitization of MMP and TIMP secretion. The positive relationship between PPBP and Vintage reflects the impact of progressive platelet dysfunction on tissue dysregulation. Figure was prepared in Matlab R2019 (Mathworks, Nattick MA, www.mathworks.com).

Platelet lysate (PL) from pre- and post-HD blood samples were assessed for relative RNA changes to examine chronic effects on platelet function between treatment sessions. PCR analysis revealed greater average RNA content in post-HD platelets for all secreted biomarkers relative to generic platelet Purinergic Receptor P2Y12 (P2RY12) (Fig. 3A). This is consistent with expected platelet turnover, increasing overall RNA reserves. However, disproportionate intra-HD changes between markers reveal selective degradation following translation^41, 42^ and upregulation in megakaryocytes prior to fragmentation^25^, neglecting endogenous uptake mechanisms^43^. Significant increases were found in 2 growth factors (EGF, PPBP), 2 clotting factors (PF4, vWF), and 1 metabolic indicator (GAPDH). Among growth factor RNAs, increased EGF and PPBP may signify enhanced regenerative and fibrotic potential, while the majority decreases observed for FGF2, PDGFB, PDGFD, and TGFβ1 signify stimulated depletion without compensatory upregulation in new platelets. Among clotting factor RNAs, significant increases measured for PF4 and vWF indicate platelet hypersensitization, while the majority decreases observed for PAF RNA may presage a pathway to long-term dysfunction. While no significant intra-HD changes were found in MMPs or TIMPs, MMP1 decreased while all 3 measured TIMP transcripts increased in a majority of patients, indicating preferential TIMP upregulation. The multi-modal distribution of TIMP1 changes reveals patient subsets exhibiting either depleted or highly upregulated TIMP1 RNA. Because of patient differences in rates of platelet renewal, this could signify that HD stimulates both TIMP1 translation in existing platelets and upregulation in subsequent platelet production. The parallel significant increase in GAPDH represents the increased metabolic capacity of platelets upon renewal. When correlated simultaneously against patient predictors by multivariate linear regression, Vintage uniquely predicted ΔPAFAH2 RNA (*p* = 1.9E−4) (Table ST6). Increasing intra-dialytic PAF upregulation (R = 0.861, *p* = 9.2E−6) (Fig. 3B) supports altered platelet translative potential leading to clotting dysfunction with long-term HD. These results imply that beyond RNA renewal effects of chronically shortened platelet life cycles, megakaryocytes may respond to iatrogenic stress by shifting platelet protective and translative behaviors.

### Platelet secretomes shift with dialysis vintage

To determine intra-dialytic effects on platelet activity, blood was re-sampled from 12 HD patients for platelet proteomic analysis (Fig. 3C). Among measured MMPs, TIMPs, and GFs for paired pre- and post-HD samples, one significant decrease was observed in connective tissue growth factor (CTGF), signifying a case in which granule-mediated secretion outpaces production. While relative platelet lysate protein changes generally showed high variability, MMP3 correlated positively to BFR (*p* = 0.046) (Table ST6), signifying net synthesis accompanying platelet renewal (Fig. 3D). TIMP2 correlated negatively to Vintage (*p* = 0.012) (Table ST6), suggesting depletion after long-term HD, while PPBP correlated positively to Vintage (*p* = 0.021) (Table ST6), revealing enhanced production of fibrotic molecules (Fig. 3E). Collectively, these suggest that faster BFR increases platelet proteolytic capabilities, and that long-term HD patients develop pro-fibrotic platelets.

### Elevated activation and inflammatory state indicate platelet dysfunction

**Figure 4:**
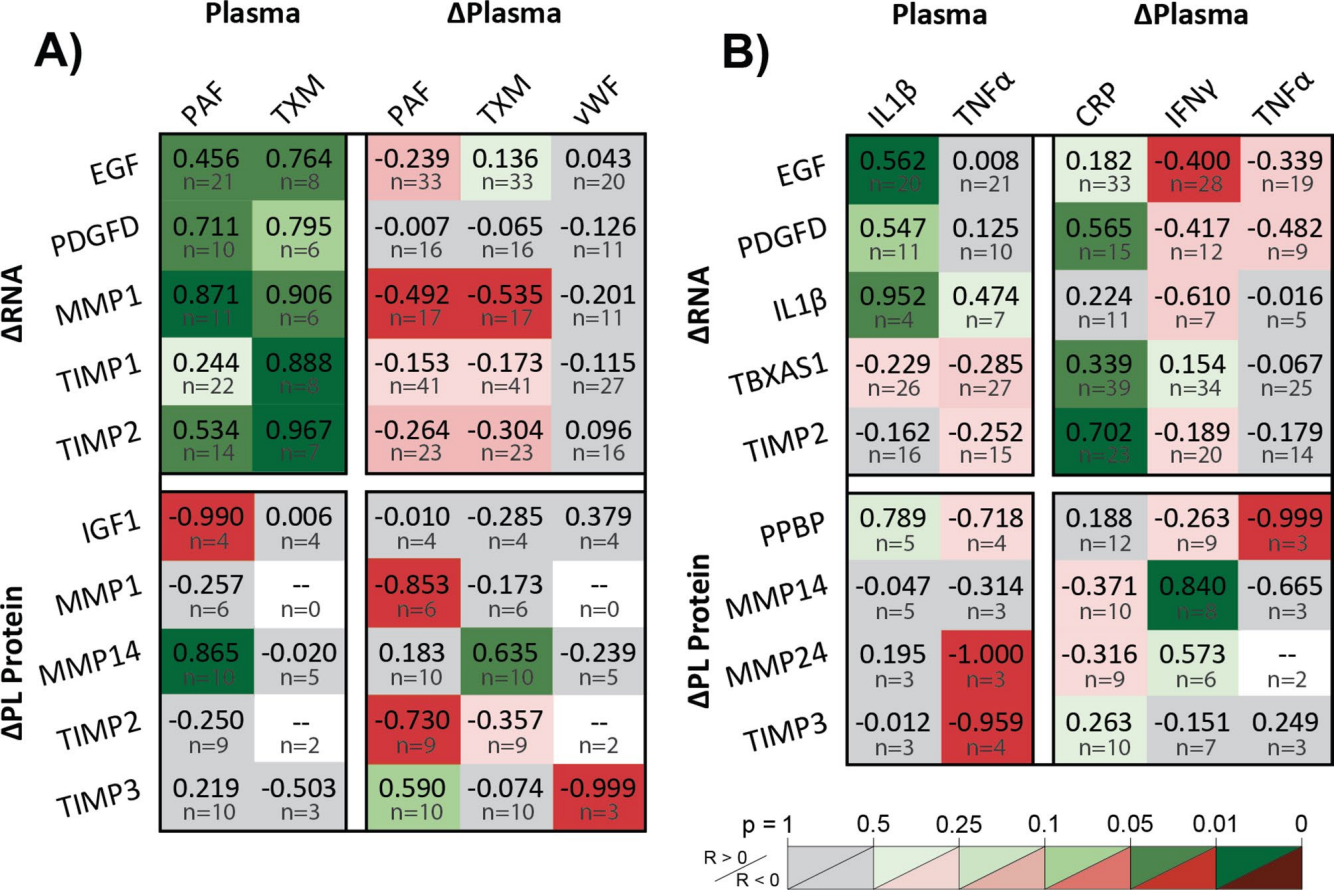
Elevated activation and inflammatory state indicate platelet dysfunction. Pearson correlation coefficients, R, of intra-dialysis platelet lysate RNA or protein relative changes against pre-dialysis plasma protein levels or intra-dialysis plasma relative changes, categorized by interpreted effects on platelets caused by (**A**) coagulative and (**B**) inflammatory indicators. (**A**) Platelet ΔRNA correlates positively to pre-dialysis levels of coagulative indicators and negatively to intra-dialysis protein changes, revealing targets of selective megakaryocyte upregulation and stimulated synthesis during dialysis. Platelet proteome correlations to plasma support acute MMP and TIMP exocytosis during dialysis and elevated chronic membrane-type MMP14 presentation with plasma levels of platelet activators. Increasing acute MMP and TIMP ΔRNA with increasing chronic plasma indicators, coupled with decreasing ΔRNA with increasing acute Δplasma indicators attributed to translation and degradation support MMPs and TIMPs as platelet-mediated targets causing dysfunction. (**B**) Platelet secretome correlations to chronic levels and acute changes in plasma inflammatory markers show platelet contributions to inflammatory and regenerative response. EGF is chronically enhanced and acutely synthesized, while IL1β is chronically amplified in platelets. Meanwhile, PDGFD, TIMP2, and TBXAS1 RNA are protected with acute inflammation. Membrane-type MMPs appear to be chronically suppressed and acutely enhanced, while TIMP3 synthesis is chronically suppressed and PPBP is acutely secreted. Figure was prepared in Matlab R2019 (Mathworks, Nattick MA, www.mathworks.com).

Plasma measurements revealed chronic accumulation of MMPs and TIMPs and acutely heightened TIMP1, while disproportionate changes in the platelet secretome revealed altered protein synthesis between HD sessions. However, further examination of platelet secretome correlations to plasma clotting, inflammatory, and cardiovascular regulators revealed fundamental behavioral adaptations to HD (Fig. 4). In aggregate, these findings reveal accelerated platelet turnover with enhanced translative capacity, augmented proteolytic enzyme translation and secretion, elevated platelet inflammatory and regenerative response, platelet dysfunction in patients with attenuated native platelet inhibition, and further evidence of worsening dysfunction with faster BFR and longer Vintage.

#### Chronic elevation of platelet activators accelerates platelet turnover and enhances overall translative capacity, while acute elevation preferentially augments MMP/TIMP translation and secretion

Platelet secretome correlations to plasma measurements of clotting factors connect platelet activation with both long- and short-term changes in transcriptomic potential and secretory behavior (Fig. 4A). Multiple positive correlations between RNA and pre-HD plasma clotting factor levels reveal selective growth factor, MMP1, and TIMP upregulation upon platelet renewal for patients with chronic elevation of PAF and TXM. In parallel, negative RNA correlations to acute plasma clotting factor changes show selective translation and degradation of MMP1 RNA with iatrogenic clotting. Negative platelet lysate IGF1 and positive MMP14 correlations to pre-HD plasma PAF reveal heighted platelet sensitization to granule secretion and membrane-MMP presentation. Finally, correlations between the change in platelet lysate (ΔPL) and plasma (Δplasma) clotting factors similarly show MMP and TIMP secretion and MMP14 presentation with acute clotting. When collectively applied as predictors of platelet activity, multivariate linear regression reveals that pre-HD clotting factor elevation of PAF and TXM indicate MMP1 upregulation (R^2^ = 0.91, *p* = 0.027), while pre-HD elevation and intra-HD clearance of PAF and TXM indicate TIMP2 upregulation (R^2^ = 0.98, *p* = 0.044) (Table ST7).

#### Inflammatory elevation accompanies amplified platelet inflammatory and regenerative response

Inflammatory cytokines influence the platelet secretome in dialysis patients (Fig. 4B). Platelet EGF and IL1β RNA increased with plasma IL1β, while EGF RNA was translated and degraded with plasma ΔIFNγ, showing regenerative and inflammatory contributions with systemic inflammation. Conversely, positive PDGF-DD, TIMP2, and thromboxane (TX) RNA correlations to acute plasma ΔCRP suggest that acute inflammation suppresses translation and degradation of pro-fibrotic RNA (R^2^ = 0.87, *p* = 0.0007) (Table ST8). Whereas clotting factor elevation predicted platelet MMP/TIMP production and secretion, inflammatory elevation predicted growth factor amplification and production. Elevated plasma TNFα accompanied reduced ΔMMP24 and ΔTIMP3, suggesting reduced MMP/TIMP production. Degranulation with acute inflammation is observed by presentation of MMP14 and depletion of PPBP.

#### Patients with higher native platelet inhibition show lower platelet dysfunction

**Figure 5:**
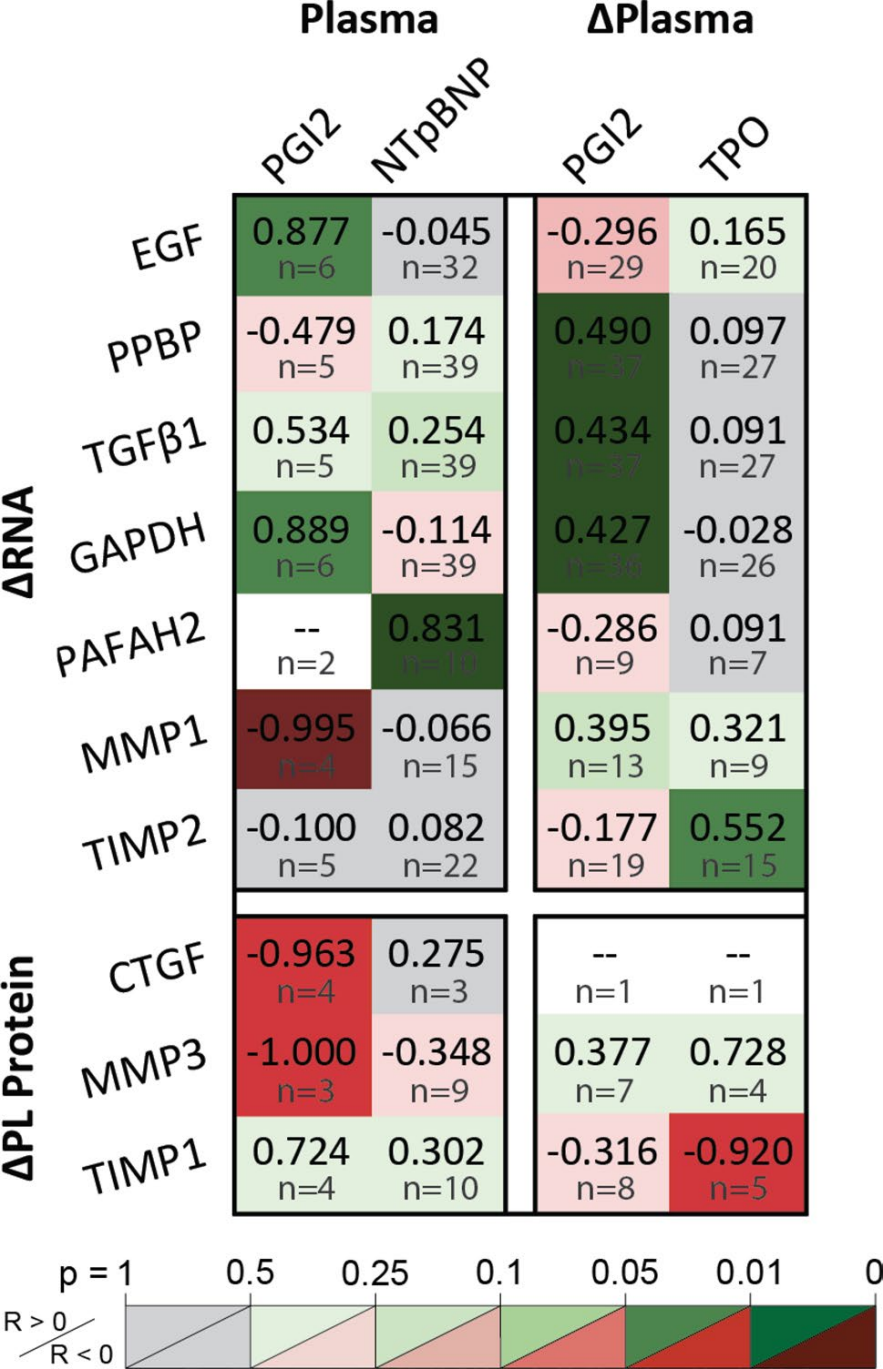
Cardiovascular stress markers indicated platelet dysfunction. Platelet inhibition by PGI_2_ protects RNA from translation as shown by positive correlations against EGF, GAPDH, PPBP, and TGFβ1 transcripts and negative correlations against granule CTGF and MMP3. PAFAH2 upregulation correlates to plasma NTpBNP, showing platelet dysfunction with chronic cardiac strain. Platelet renewal is shown by TIMP2 RNA increases and granule TIMP1 decreases with plasma TPO. Figure was prepared in Microsoft Excel v2020 (Microsoft Corp. www.microsoft.com).

Platelet correlations to cardiovascular markers validate known regulatory and dialytic effects (Fig. 5). Platelet dysfunction is observed by increasing PAF RNA with plasma NTpBNP. TIMP2 RNA upregulation and PL TIMP1 depletion with plasma ΔTPO shows selective TIMP renewal with platelet turnover. Positive RNA correlations to plasma PGI2 and ΔPGI2 validate platelet inhibition of RNA translation and degradation, though minimal improvement in adjusted R^2^ is observed by multiplexing RNA predictors against plasma PGI2 (Table ST9). Finally, platelet MMP1 RNA, MMP3 and CTGF production are attenuated by higher plasma PGI2. Thus, normal restorative platelet inhibition indicates lower platelet dysfunction.

#### Faster BFR and longer HD Vintage exacerbate platelet dysfunction

Comparisons of platelet RNA and protein levels against clotting factors indicate broader effects of BFR and Vintage on long-term platelet translation and secretion. Faster BFR increasingly stimulates platelet dysfunction, shown by comparison of pre-HD plasma PAF to BFR (Fig. 1D) by multivariate linear regression. Similarly, stimulation of platelet RNA renewal and degranulation with plasma clotting factor elevation depict a mechanism of increasingly stimulated platelet dysfunction with BFR (Fig. 4A). Decreasing plasma ΔPAF and ΔTXM with both higher BFR and Vintage, respectively (Fig. 2B, C), and increasing ΔMMP1 RNA and PL ΔMMPs and ΔTIMPs with decreasing ΔPAF and ΔTXM (Fig. 4A) show preferential augmentation of platelet MMPs and TIMPs with increasing BFR and Vintage. Further, platelet-driven mechanisms causing tissue dysregulation appear to extend between HD sessions, shown by the significant relative increases in clotting factor RNA post-HD. Thus, in addition to directly enhancing platelet PAF RNA (Fig. 3B), faster BFR and longer Vintage may cause platelet behavioral adaptations that reduce regenerative contributions and disrupt proteolytic balance.

### Hemodialysis shifts platelet function from regenerative to pro-fibrotic

Platelet correlations to circulating clotting, inflammatory, and cardiovascular factors indicate worsening dysfunction with accelerated platelet turnover, subdued acute phase response, cardiac stress markers, and attenuated platelet inhibition. In parallel, secretome correlations to fibrotic and regenerative factors reveal distinct modes of platelet behavior and suggest a chronic shift toward a pro-fibrotic mileu. Increasingly pro-fibrotic platelets with proteolytic circulation and decreasingly pro-fibrotic platelets with regenerative circulation show two sides of the same coin. Regenerative feedback responses to fibrotic activity and vice versa show short-term mode-switching between HD sessions. Inverse proportionalities between heterogenous granule proteins and proteases illustrate a long-term mechanism accounting for shifts in production.

#### Perturbed plasma proteolytic balance is associated with pro-fibrotic platelets with enhanced MMP/TIMP production and secretion

**Figure 6:**
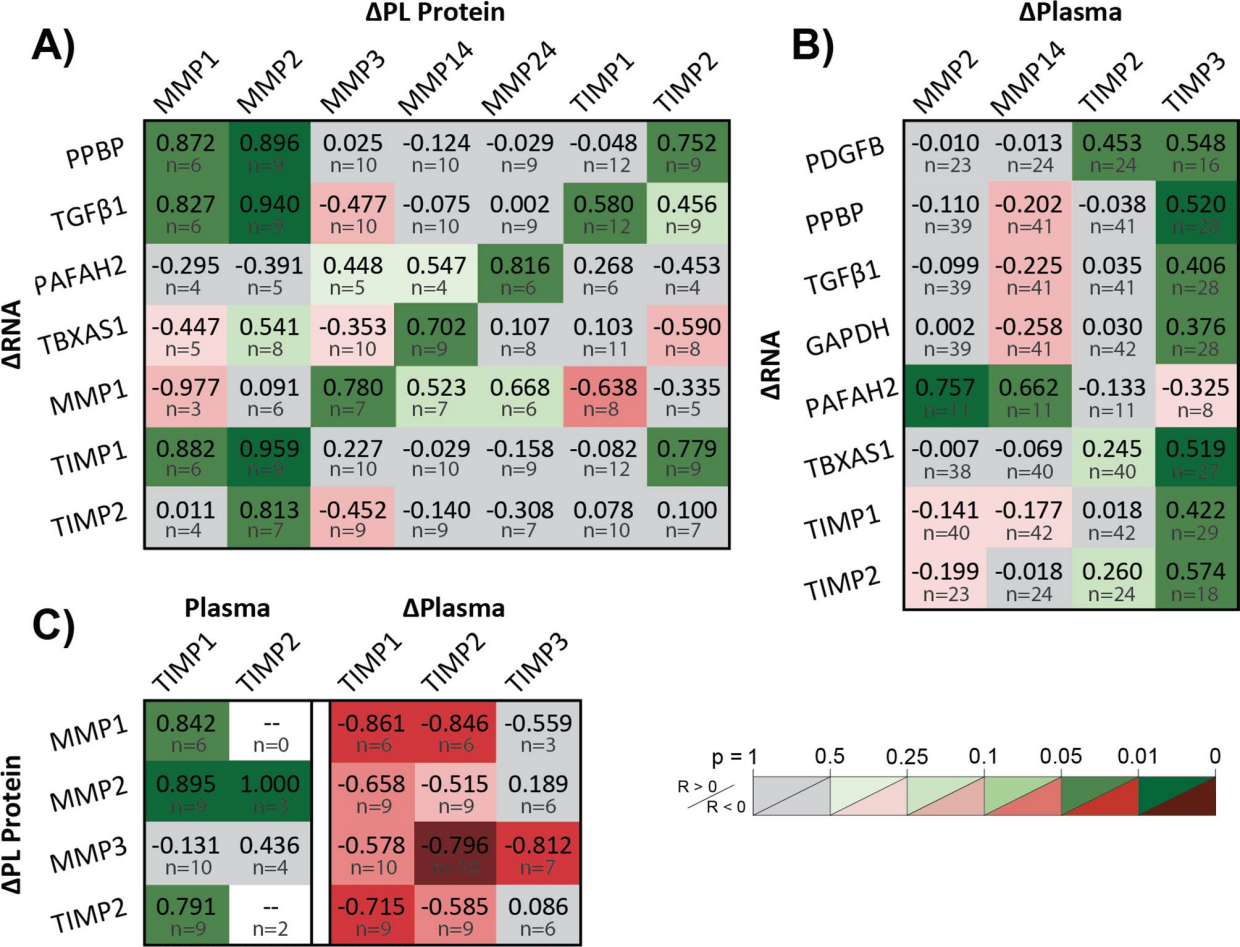
Pro-fibrotic platelet secretomes correlate to elevated plasma MMPs and TIMPs. Pearson correlation coefficients, R, of fibrotic platelet (**A**) ΔRNA against platelet ΔMMPs and ΔTIMPs, (**B**) ΔRNA against plasma ΔMMPs and ΔTIMPs, and (**C**) Δprotein against plasma TIMPs and ΔTIMPs. (**A**) Platelets with acutely enhanced MMP and TIMP secretomes have amplified transcripts for MMPs, TIMPs, platelet activators, and other fibrotic factors. (**B**) Patients presenting acute plasma MMP and TIMP increases have platelets exhibiting greater RNA for fibrotic factors. (**C**) Chronic plasma TIMP elevation accompanies greater platelet production of MMPs and TIMPs, and increased intra-dialysis MMP and TIMP secretion. Figure was prepared in Microsoft Excel v2020 (Microsoft Corp. www.microsoft.com).

Increasing granule MMPs and TIMPs with increasing pro-fibrotic RNA identify platelet populations with enhanced fibrotic contributions (Fig. 6A). Granule MMPs and TIMPs increased with fibrotic GF, MMP, and TIMP RNA, while membrane-type MMPs increased with clotting factor RNA. Platelet TGFβ1, TIMP1, and PPBP upregulation predicted stored MMP1 (R^2^ = 0.88, *p* = 0.043) and MMP2 (R^2^ = 0.92, *p* = 0.0033) concentrations (Table ST10). Increasingly pro-fibrotic platelet transcriptomes are also identified by acute plasma MMP and TIMP increases (Fig. 6B). Fibrotic GF, TIMP, and TX transcripts increased with acute increases in plasma TIMPs (R^2^ = 0.45, *p* = 0.025) (Table ST11), while PAF RNA increased with acute increases in plasma MMPs (R^2^ = 0.60, *p* = 0.026) (Table ST12). The self-amplifying cycle of fibrotic transcript upregulation and protein synthesis with acute proteolytic imbalance is further supported by observations of enhanced platelet MMP/TIMP production and secretion (Fig. 6C), shown by increases in platelet lysate MMP/TIMP with chronic plasma TIMP elevation (R^2^ = 0.79, *p* = 0.043) and decreases in platelet lysate MMP/TIMP with acute plasma TIMP elevation (R^2^ = 0.79, *p* = 0.043) (Table ST13).

#### Higher plasma levels of fibro-protective GFs correlate to lower fibrotic and higher regenerative contributions by platelets

**Figure 7:**
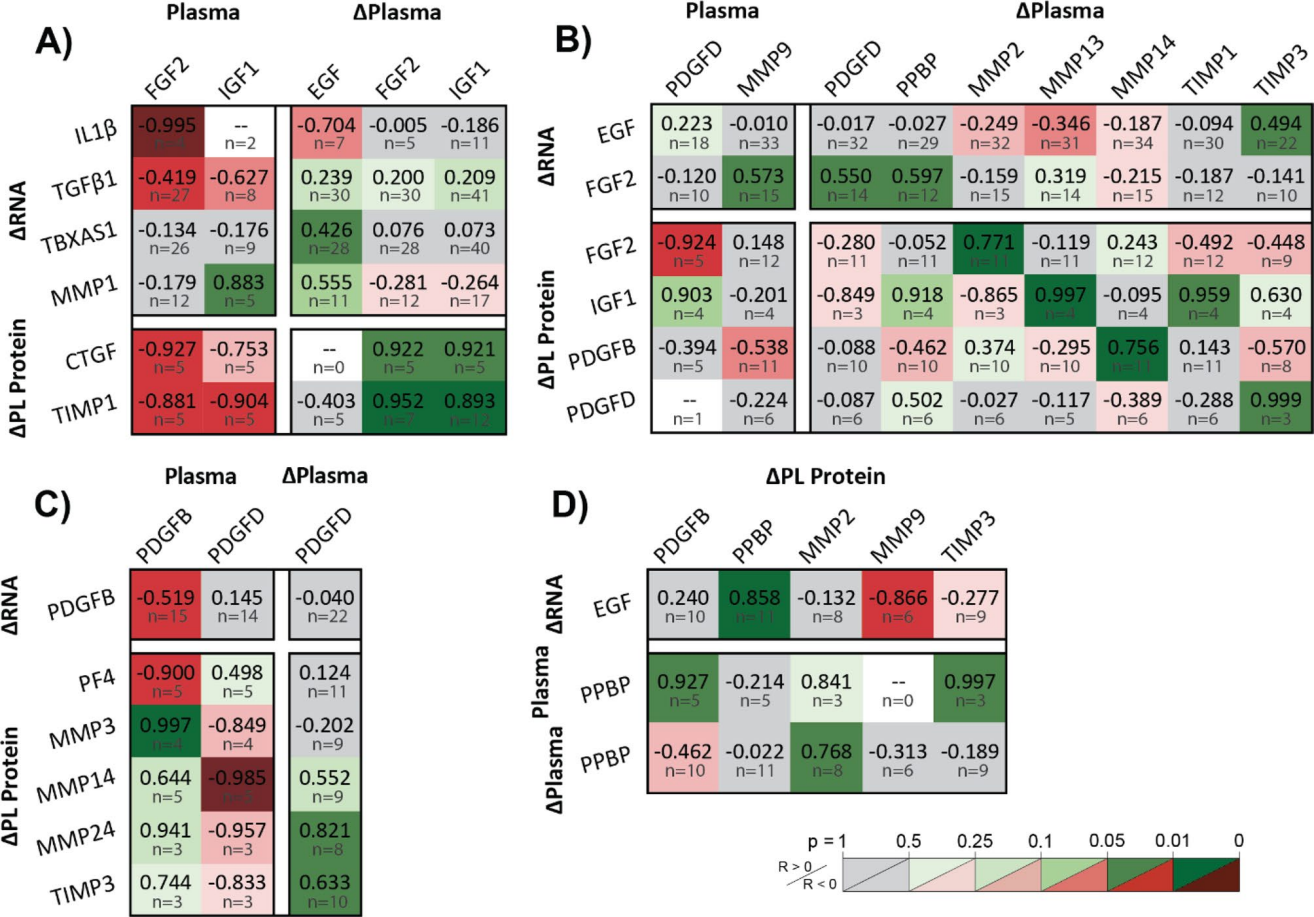
Growth factor and proteolytic trends suggest a chronic shift in platelet function from regenerative to pro-fibrotic. Pearson correlation coefficients, R, of intra-dialysis platelet lysate RNA or protein relative changes against pre-dialysis plasma protein levels or intra-dialysis plasma relative changes, categorized by interpreted effects on platelets caused by (**A**) growth factors, (**B**) fibrotic activity, and (**C**) PDGFs. (**A**) Higher plasma levels of regenerative growth factors indicated platelets with decreasing fibrotic contributions. Chronic fibro-protective FGF2 plasma elevation correlated to lower fibrotic molecule production and RNA, and acute plasma FGF2 elevation correlated to fibrotic molecule retention. Plasma EGF reduces TBXAS1 translation, while IGF1 shows mixed effects including MMP1 amplification. (**B**) Compensatory mechanisms are observed by acute enhancement of regenerative factors with acute plasma fibrotic activity, showing transient mode-switching between dialysis sessions. Growth factor RNA and protein synthesis accompany acute plasma MMP and TIMP increases. Plasma PDGFD elevation accompanies FGF2 RNA upregulation and protection. (**C**) PDGF feedback shows an example of fibrotic enhancement of regenerative potential. PDGFB RNA decreases with increasing plasma PDGFB levels, showing degradation after translation and secretion. Rising PDGF levels accompany decreased granule PF4, TIMP3 and MMP3 retention, and acutely stimulated presentation of membrane-MMPs. (**D**) Correlations among alpha-granule proteins and proteases elucidate physiological adaptations contributing to pro-fibrotic or fibro-protective functions. Decreasing MMP9 and increasing PPBP in platelets with increasing EGF RNA show a trade-off in differential alpha-granule filling behaviors. Acute plasma PPBP elevation, which showed to accompany regenerative RNA enhancement, correlated to MMP2 retention. Figure was prepared in Microsoft Excel v2020 (Microsoft Corp. www.microsoft.com).

Higher plasma levels of regenerative growth factors indicated platelets with decreasing fibrotic contributions (Fig. 7A). Plasma FGF2 correlated to reduced RNA for inflammatory IL1β and fibrotic TGFβ1.Platelet production and secretion of fibrotic CTGF and TIMP1 decreased with plasma FGF2 and IGF1, indicated by negative correlations to pre-HD levels and positive correlations to intra-HD changes. Using MLR, TGFβ1 RNA retention and reduced PL CTGF and TIMP1 predicted plasma FGF2 levels (R^2^ = 1.0, *p* = 0.0021) (Table ST14). Further, platelet TIMP1 was predicted by plasma IGF1 depression pre-HD, accumulation intra-HD, and FGF2 clearance (R^2^ = 1.0, *p* = 0.036) (Table ST14). However, unlike FGF2, plasma IGF1 correlated to increased platelet MMP1 RNA. Finally, clotting factor translation was reduced with ΔEGF, shown by TX RNA preservation.

#### Acute elevation in fibrotic activity and PDGFs accompany a regenerative platelet response between treatment sessions

Acute enhancements in platelet secretome growth factors to acute plasma increases in fibrotic activity suggest short-term behavioral adaptations between HD sessions (Fig. 7B). Increased fibro-protective RNA (FGF2 and EGF) with fibrotic plasma (MMP9, ΔPPBP, and ΔTIMP3) shows a regenerative shift post-HD. This is supported by platelet IGF1 and PDGF retention with plasma ΔMMPs and ΔTIMPs. FGF2 upregulation with ΔPDGFD and retention with pre-HD PDGFD demonstrate a case of fibrotic enhancement of regenerative potential. In fact, multivariate linear regression revealed that plasma MMP9, PDGFD, and PPBP positively predict FGF2 upregulation (R^2^ = 0.76, *p* = 0.015) (Table ST15). Further feedback activity is observed by secretome correlations to PDGFs (Fig. 7C). As expected, PDGFB RNA decreases as plasma levels increase because of translation followed by degradation. Other platelet lysate correlations to plasma PDGFs show platelet depletion of PF4, retention of MMP3 and TIMP3, and acutely stimulated production of membrane-type MMPs.

#### Secretome correlations support pro-fibrotic physiological adaptations in platelets

Platelet behavioral adaptations may be explained in part by selective differences in granule activity (Fig. 7D). Decreasing platelet lysate MMP9 and increasing platelet lysate PPBP with increasing EGF RNA show an inverse trade-off between producing proteases and other alpha granule proteins. Further, plasma PPBP was accompanied by reduced protease secretion, observed by platelet lysate MMP2 retention, and increased alpha-granule production elsewhere, observed by TIMP3 and PDGFB synthesis. Thus, increasingly regenerative platelet phenotypes may be characterized by disproportionate growth factor accumulation and protease attenuation. Conversely, increasingly fibrotic platelets may present with protease accumulation and heterogenic alpha granules with pro-fibrotic disproportion.

## Discussion

Many plasma biomarker measurements reported in this study mirror prior findings in HD patients demonstrating effects of uremia^10, 18, 19, 22^, acute inflammatory response^30–32^, cardiovascular risk through mechanical strain and oxidative stress^33, 44^, coagulative dysfunction^26^, and microvascular damage and systemic fibrosis^28, 29, 34–38, 45^. Comparison of these measurements to prescribed dialysis metrics and quantitative platelet characterization yield novel insights into a circulatory environment in HD patients that is characterized by profound shifts in ECM homeostasis, microvascular damage, and platelet function. First, chronic plasma elevation of MMPs and TIMPs, insufficient removal by dialysis, and disproportionate acute TIMP1 enhancement characterize dysregulated ECM homeostasis. These symptoms accompany enhanced production and secretion of MMPs and TIMPs by platelets, illustrating a vicious cycle of dysregulation. Second, depressed FGF2^34, 45^, elevated IGF1^35^, and elevated PDGFD^37^ in plasma indicate high risk for microvascular damage and fibrosis, supported by increasing platelet contributions of pro-fibrotic TIMP1, CTGF, and TGFβ1 with decreasing plasma FGF2 and increasing MMP1 RNA with plasma IGF1. Third, platelet secretome correlations reveal adaptive mechanisms that enhance cardiovascular risk. Elevated NTpBNP, which implies cardiovascular strain, indicated greater platelet coagulation, while elevated clotting markers accompanied preferential MMP and TIMP translation and secretion. This pro-fibrotic platelet dysfunction decreased with native platelet inhibition, increasing inflammatory response, and regenerative GF circulation. Contrarily, acute increases in plasma fibrotic activity and PDGFs elicited a reduced pro-fibrotic platelet response in the short-term. Finally, differences between rates of platelet-released factors demonstrate chronic adaptations affecting differential secretory kinetics of alpha-granule subtypes, known to be heterogenous and specialized for inflammatory, immune, and tissue regulatory functions beyond hemostasis^46^.

Heightened BFR has been linked to increased HD patient hospitalization and mortality in studies comparing dialysis service providers and national standards^47, 48^, however underlying mechanisms are generally unexplored. The correlations observed in this study between platelet function, HD filtration rate and secondary effects of high-BFR reveal potential deregulatory mechanisms of increasing BFR. HD patients prescribed higher BFR had higher pre-HD plasma PAF and MMP3 levels despite higher post-HD clearance of PAF, MMP2, and MMP3. In platelets, higher BFR enhanced both PAF RNA and intracellular MMP3. Consequently, PAF and MMP enhancement in plasma correlated to higher overall platelet RNA with emphasis on fibrotic transcripts and augmented MMP translation and secretion. Similar trends were observed against HD Vintage, with increasing PAF RNA, PPBP synthesis, and platelet MMP1 and TIMP2 secretion over time. Thus, while coagulative reactivity has been shown to remain consistent throughout individual HD sessions^49^, platelet behaviors beyond coagulation may naturally change with longer prior time on dialysis, and increasing BFR may exacerbate pathogenesis by accentuated repetitive and excessive stimulation of platelets during therapy. Increasing plasma PAF with BFR and platelet RNA content may be of particular significance in driving microvascular damage not only in the direct promotion of leukocyte activation^38^, but also indirectly by bridging leukocyte-endothelial adhesion by P-selectin presentation which is enhanced by degranulation^39^. Chronic change in platelet function considering no acute change in coagulation may in part be explained by receptor shedding, given long-term accumulation of MMPs in spite of intra-HD clearance^50^. Importantly, pharmacological targeting of the sequalae of endothelial dysfunction including multi-organ fibrosis^14, 51–53^ (NCT02285920) and mitochondrial dysfunction^54, 55^ is an emerging paradigm to reduce cardiovascular mortality among HD patients. Our data suggest that HD-induced dysfunctional platelets likely contribute to the milieu of imbalanced plasma growth factors and proteolytic dysregulation that influence remodeling of endothelium and ECM in ESRD patients.

This study utilized a small patient sample size and did not examine whether similar changes are observed in CKD patients not on dialysis, or those on peritoneal dialysis. While these would have been interesting avenues to explore, the Covid-19 pandemic limited the ability to acquire additional data from this high-risk patient population. High variability in data sample sizes particularly for platelet transcriptome and proteome biomarkers, measurement distributions in Pearson correlations not spread widely over one axis, and ethnic incongruity between control and patient demographics may limit validity of deductions. Further, the large range of prior times on dialysis may introduce variability in acute biological response owing to the degree of tissue changes that may have already occurred. Future studies could provide greater mechanistic insight by performing media transfer experiments to elucidate platelet-leukocyte interactions. Importantly, these findings suggest that further research into whether methods to reduce platelet dysfunction in HD combined with pharmaceutical therapy using the aforementioned emerging treatments is needed in order to draw mechanistic conclusions.

## Conclusion

The findings of this study support the emerging potential of platelet contributions to tissue proteolytic dysregulation that may contribute to multi-organ fibrosis and overall cardiovascular decline following HD initiation. Repetitive and excessive platelet activation, exacerbated by higher BFR and Vintage, causes plasma regulatory imbalances by platelet dysfunction, observed by disproportionate transcriptomic upregulation and shifts in synthesis and secretion. Emphasis on MMP and TIMP activity demonstrates a potential causal link whereby platelet dysfunction systemically dysregulates tissue maintenance. Prolonged residence of platelet-released compounds, shortened platelet half-lives, and adaptive secretome enhancements between HD sessions illustrate a vicious cycle of increasingly deregulated platelet circulatory contributions. When integrated over repeated treatments, these potentially participate in the pathogenesis of systemic fibrosis and microvascular disease. The described mechanisms suggest that further investigation is warranted into the role of, and potential treatments to target, platelet contributions to dysregulated tissue maintenance in HD patients.

## Materials and methods

### Study recruitment

HD blood samples were collected immediately before vascular access and after tubing removal from patients at DaVita facilities in Berkeley and Oakland. All patients were dialyzed using NIPRO single-use, hollow-fiber ELISIO filters. Control blood samples from healthy adults with no history of smoking, drug abuse, current medications, or comorbidities were drawn at the University of California, Berkeley Tang Center. Given variability between manufacturers of ELISA tests, control samples were used to provide ranges of normal values for each assay. Patient samples were processed immediately after HD sessions, and control samples were processed immediately after collection. Demographics, documented causes of renal failure, comorbidities, and comedications are summarized in Tables ST1 and ST2. No differences were found among plasma or platelet measurements owing to ethnicity or vascular access as assessed by ANOVA. Participants provided informed consent under protocols approved by the U.C. Berkeley IRB and in compliance with the Declaration of Helsinki. All methods were performed in accordance with the relevant guidelines and regulations therein.

### Plasma and platelet isolation

Blood samples were collected in ACD-A Vacutainers and sterile-processed by combination with inhibitor cocktail (20 µM PGE1, 6 mM acetylsalicylic acid, and 400 mM EDTA at 1:200 dilution), centrifugation at 200 g, and drip filtration through an Acrodisc WBC Isolation Syringe filter. Remaining erythrocyte and leukocyte contamination was immunoprecipitated by anti-CD235a and anti-CD45 conjugated Dynabeads. Platelets and plasma were separated by centrifugation at 2000 g. Plasma was aliquoted and stored at − 80 °C for up to 1 month. Platelets were lysed in Trizol and stored at − 80 °C for up to 1 month.

### Enzyme immunoassay kits for plasma proteomics

Plasma protein concentrations were measured using pre-made ELISA and Luminex assay kits per manufacturers’ directions (Table ST3). Samples were tested in duplicate within the same assay, leading to exclusion of measurements with CV% greater than 30%. Three plates were used per marker. Some samples were re-tested across assay kits for validation. Entire assay kits were excluded from control-HD comparison based on a combination of CV% of inter-assay replicates, three-sample Kruskal–Wallis, and two-sample Kolmogorov–Smirnov tests (*p* ≤ 0.05). Absorbance measurements were interpolated to a 5-parameter logistic standard curve using Matlab. For intra-HD change analysis, if either pre- or post-measurement was invalidated or fell outside of test ranges, both measurements were excluded.

### Quantitative PCR for platelet lysate

Platelet RNA was extracted from Trizol by phase-separation with chloroform and precipitation by isopropanol. The RNA pellet was washed with 75% ethanol, reformed by centrifugation (7500 g/4 °C), and dissolved in nuclease-free water after decanting. Samples were purified using Qiagen RNA Cleanup Kits. cDNA was synthesized using Superscript IV Vilo Master Mix according to RNA concentrations measured by Qubit Fluorometer and probed using TaqMan primers (Table ST4), with TaqMan Fast Advanced Master Mix. Primer amplification was performed in a QuantStudio 3 Real-Time PCR System. Paired PCR samples with Ct values above 38 were excluded.

### Western blots for platelet lysate

Protein was precipitated from Trizol using acetone at − 20 °C followed by centrifugation (12000 g/4 °C). The protein pellet was washed with 0.3 M guanidine hydrochloride in 95% ethanol and 100% ethanol before dissolution in 3% SDS in ultrapure water. Solubilized protein was aliquoted and stored for single use at − 20 °C for up to 1 month, according to concentrations measured by BCA assay. Protein samples were denatured by LDS and reduced, electrophoresed at 20 µg loads, and transferred to PVDF membranes by Power Blotter. Membranes were probed for one target by iBind Flex, stripped and re-probed for loading control, and discarded. Target bands were detected using SuperSignal West Pico, SuperSignal West Femto, and SuperSignal Enhancer where appropriate. Membranes were imaged using a ChemiDoc XRS. Band densitometry was performed using ImageJ. Antibodies used are listed in Table ST5.

### Statistics

HD versus control plasma measurement distributions were compared by two-sample t test, assuming unequal variance and two tails. Intra-HD plasma relative protein changes, platelet RNA changes, and PL protein changes were assessed by one-sample t test using two tails. Linear dependencies were assessed by multiple linear regression (MLR), and unique linear dependencies to individual predictors were assessed by Pearson correlation. Figures 4, 5, 6, and 7 tabulate correlation coefficients for manually grouped comparisons and are automatically colored according to predictive strength *p*, with raw data plots for significant correlations depicted in Figs. SF4, SF5, and SF6. Significance in all cases was determined by *p* ≤ 0.05. Figure error bars represent 95% confidence intervals. Box and whisker plots represent sample quartiles.

## Supplementary Information

Supplementary Information 1.

## Data Availability

For deidentified original data, please contact moriel@berkeley.edu.
