# Supplementary material for: Hemodialysis exacerbates proteolytic imbalance and pro-fibrotic platelet dysfunction
Source: Sci Rep. 2021 Jun 3;11:11764. doi: 10.1038/s41598-021-91416-8 (PMC8175411; doi:10.1038/s41598-021-91416-8)
Supplement: Supplementary file 1 — Supplementary Information 1. [file 41598_2021_91416_MOESM1_ESM.docx]

Hemodialysis exacerbates proteolytic imbalance and pro-fibrotic platelet dysfunction

Short title: Pro-fibrotic platelet modulation in hemodialysis

Authors: Aaron J Velasquez-Mao^1^, Mark A Velasquez^2^, Zhengxiong Hui^2^, Denise Armas-Ayon^2^, Jingshen Wang^3^, Moriel H Vandsburger^2^.

^1^ UC Berkeley-UCSF Graduate Program in Bioengineering

^2^ Department of Bioengineering, UC Berkeley

^3^ Department of Biostatistics, UC Berkeley

Corresponding Author:

Moriel Vandsburger, PhD

281 Hearst Memorial Mining Building

U.C. Berkeley

Berkeley, CA

94721

USA

1-510-664-9679

moriel@berkeley.edu

**Supplemental Materials**

**Supplemental Table ST1. Dialysis patient demographics.**

| Table 1. Patient demographics (n=56) | |
| --- | --- |
| Age, years - median (range) | 61.905 (23.83-85.25) |
| Gender ratio - [male/female] | 32/24 |
| Pre-Dialysis Weight, kg - median (range) | 79.65 (49.6-160.7) |
| Post-Dialysis Weight, kg - median (range) | 78.35 (48.7-158.9) |
| Height, cm - median (range) | 167.65 (150-185.5) |
| Ethnicity |  |
| African American | 29 |
| Asian American | 7 |
| Caucasian | 13 |
| Hispanic | 7 |
| Prior time on dialysis, months - median (range) | 39.417 (1.97-221.77) |
| Filter surface area, m2 - [1.5/1.7/1.9] | 22/29/5 |
| Vascular Access - [shunt/fistula/catheter] | 14/33/9 |
| Cause of renal failure |  |
| Hypertension | 37 |
| Diabetes mellitus | 25 |
| Polycystic kidney disease | 1 |
| IgA Nephropathy | 1 |
| Analgesics | 0 |
| MPGN | 0 |
| Unknown | 14 |
| Comorbidities |  |
| Anemia | 16 |
| Hyperlipidemia | 10 |
| CHF | 7 |
| Hepatitis | 6 |
| Hypothyroidism | 5 |
| Medications |  |
| Lipid-lowering drugs | 20 |
| Platelet inhibitor | 12 |
| RAS Inhibitors | 10 |
| Coumarin derivatives | 2 |
| None | 11 |
| Unknown | 15 |

**Supplemental Table ST2. Healthy control demographics.**

| ST2. Control demographics (n = 25) | |
| --- | --- |
| Age, years - median (range) | 53.9 (45.3-76.2) |
| Gender ratio - [male/female] | 16/9 |
| Weight, kg - median (range) | 71.2 (45.4 - 113.4) |
| Height, cm - median (range) | 175 (155 - 188) |
| Ethnicity |  |
| African American | 0 |
| Asian American | 5 |
| Caucasian | 18 |
| Hispanic | 2 |

**Supplemental Table ST3. Enzyme immunoassay kits used to for plasma measurements.**

| **Protein** | **Classification** | **Manufacturer** | **Detection range** | **Dilution** |
| --- | --- | --- | --- | --- |
| 11TXB2 | Cardiovascular indicator | Elabsci (E-EL-H0207) | 15.6-1,000 pg/mL | 1:1, 1:6 |
| CRP | Inflammatory indicator | ThermoFisher (EPX01A-10288-901) | 5.66-5800 pg/mL | 1:500 |
| CTGF | Growth factor | Cloud-Clone (SEA010Hu) | 7.8-500 pg/mL | 1:1, 1:2 |
| EGF | Growth factor | Cloud-Clone (SEA560Hu) | 3.9-250 pg/mL | 1:2, 1:4 |
| FGF2 | Growth factor | Cloud-Clone (CEA551Hu) | 12.4-1,000 pg/mL | 1:1 |
| GAPDH | Cardiovascular indicator | ThermoFisher (EPX01A-12154-901) | 244-250,000 pg/mL | 1:1 |
| IFNγ | Inflammatory indicator | ThermoFisher (EPX01A-10228-901) | 12-11,925 pg/mL | 1:1 |
| IGF1 | Growth factor | Cloud-Clone (SEA050Hu) | 156-10,000 pg/mL | 1:50, 1:100 |
| IL1β | Inflammatory indicator | ThermoFisher (EPX01A-10224-901) | 2.14-8,750 pg/mL | 1:1 |
| LAP (TGFβ1) | Growth factor | ThermoFisher (BMS2065) | 156-10,000 pg/mL | 1:2.5, 1:25 |
| MMP2 | Proteolytic regulation | ThermoFisher (EPX03A-10829-901) | 36-146,700 pg/mL | 1:25, 1:50 |
| MMP3 | Proteolytic regulation | ThermoFisher (EPX03A-10829-901) | 18-18,125 pg/mL | 1:25, 1:50 |
| MMP9 | Proteolytic regulation | ThermoFisher (EPX03A-10829-901) | 0.72-2,950 pg/mL | 1:25, 1:50 |
| MMP13 | Proteolytic regulation | ThermoFisher (EPX01A-12131-901) | 7.08-29,000 pg/mL | 1:1 |
| MMP14 | Proteolytic regulation | Wuhan (EH0369) | 31-2,000 pg/mL | 1:2, 1:6 |
| NTpBNP | Cardiovascular indicator | Wuhan (EH0350) | 39-2,500 pg/mL | 1:1, 1:2 |
| PAF | Clotting Factor | Elabsci (E-EL-H2199) | 78.1-5,000 pg/mL | 1:6 |
| PDGFB | Growth factor | RayBiotech (ELH-PDGFBB-1) | 1-400 pg/mL | 1:1, 1:2 |
| PDGFD | Growth factor | Cloud-Clone (SEC919Hu) | 12.5-800 pg/mL | 1:1 |
| PF4 | Clotting Factor | ThermoFisher (EHPF4) | 20.58-15,000 pg/mL | 1:100, 1:200 |
| PGI2 | Cardiovascular indicator | Blue Gene (ABIN771046) | 25-500 pg/mL | 1:1 |
| PPBP (βTG) | Growth factor | ThermoFisher (EHPPBP) | 4.1-1,000 pg/mL | 1:600, 1:5,000 |
| TIMP1 | Proteolytic regulation | ThermoFisher (EPX01A-12018-901) | 146-150,000 pg/mL | 1:20, 1:50 |
| TIMP2 | Proteolytic regulation | ThermoFisher (EHTIMP2) | 1.6-1,200 pg/mL | 1:300 |
| TIMP3 | Proteolytic regulation | Cloud-Clone (SEA129Hu) | 150-10,000 pg/mL | 1:1, 1:4 |
| TNFR1 | Inflammatory indicator | ThermoFisher (EPX01A-10203-901) | 189-774,400 pg/mL | 1:1 |
| TPO | Cardiovascular indicator | ThermoFisher (EPX01A-12161-901) | 94-96,275 pg/mL | 1:1 |
| vWF | Clotting Factor | ThermoFisher (EPX040-10825-901) | 0.00244-2.5 %plasma | 1:500 |

*Kits not purchased from ThermoFisher were purchased through Antibodies-Online.

**Supplemental Table ST4. Primers used for PCR.**

| **RNA Target** | **Details** | **Accession #** | **Assay ID (Thermo)** |
| --- | --- | --- | --- |
| EGF | Growth factor | NM_001178130.2, NM_001178131.2, NM_001963.5 | Hs01099990_m1 |
| FGF2 | Growth factor | NM_002006.4 | Hs00266645_m1 |
| GAPDH | Metabolic indicator | NM_002046.5, NM_001256799.2, NM_001289745.1, NM_001289746.1 | Hs02786624_g1 |
| IFNγ | Inflammatory cytokine | NM_000619.2 | Hs00989291_m1 |
| IGF1 | Growth factor | NM_000618.4, NM_001111283.2, NM_001111284.1 | Hs01547656_m1 |
| IL1β | Inflammatory cytokine | NM_000576.2 | Hs01555410_m1 |
| MMP1 | Proteolytic regulation | NM_001145938.1 NM_002421.3 | Hs00899658_m1 |
| MMP2 | Proteolytic regulation | NM_004530.5, NM_001127891.2, NM_001302508.1, NM_001302510.1, NM_001302509.1 | Hs01548727_m1 |
| MMP3 | Proteolytic regulation | NM_002422.4 | Hs00968305_m1 |
| MMP9 | Proteolytic regulation | NM_004994.2 | Hs00957562_m1 |
| MMP13 | Proteolytic regulation | NM_002427.3 | Hs00942584_m1 |
| MMP14 | Proteolytic regulation | NM_004995.3 | Hs01037003_g1 |
| MMP24 | Proteolytic regulation | NM_006690.3 | Hs00198580_m1 |
| P2RY12 | Loading Control | NM_022788.4 NM_176876.2 | Hs01881698_s1 |
| PAFAH2 | Clotting factor | NM_000437.3 | Hs00166473_m1 |
| PDGFB | Growth factor | NM_002608.3, NM_033016.3 | Hs00966522_m1 |
| PDGFD | Growth factor | NM_025208.4, NM_033135.3 | Hs00228671_m1 |
| PF4 | Clotting factor | NM_002619.3 | Hs00427220_g1 |
| PPBP | Growth factor | NM_002704.3 | Hs00234077_m1 |
| TBXAS1 | Clotting factor | NM_001061.4, NM_030984.3, NM_001130966.2, NM_001166253.1, NM_001166254.1, NM_001314028.1 | Hs01022706_m1 |
| TGFβ1 | Growth factor | NM_000660.5 | Hs00998133_m1 |
| TIMP1 | Proteolytic regulation | NM_003254.2 | Hs01092512_g1 |
| TIMP2 | Proteolytic regulation | NM_003255.4 | Hs00234278_m1 |
| TIMP3 | Proteolytic regulation | NM_000362.4 | Hs00165949_m1 |
| TNFα | Inflammatory cytokine | NM_000594.3 | Hs00174128_m1 |
| vWF | Clotting factor | NM_000552.4 | Hs01109446_m1 |

Gray = Ct values detected were above cutoff threshold.

**Supplemental Table ST5. Antibodies used for WB.**

| **Protein Target** | **Classification** | **Manufacturer** |
| --- | --- | --- |
| CTGF | Pro-fibrotic | ThermoFisher (MA5-26817, 31430) |
| EGF | Fibro-protective | ThermoFisher (M805, 31430) |
| FGF2 | Fibro-protective | ThermoFisher (MA5-15276, 31430) |
| GAPDH | Metabolic activity | ThermoFisher (MA5-15738, 31430) |
| IGF1 | Pro-fibrotic | ThermoFisher (MA1-088, 31430) |
| LAP (TGFβ1) | Pro-fibrotic | ThermoFisher (MA5-17186, 31430) |
| MMP1 | Proteolytic regulation | ThermoFisher (MA1-771, 31430) |
| MMP14 | Proteolytic regulation | ThermoFisher (PA5-13183, 31460) |
| MMP2 | Proteolytic regulation | ThermoFisher (MA1-772, 31430) |
| MMP24 | Proteolytic regulation | ThermoFisher (PA5-51333, 31460) |
| MMP3 | Proteolytic regulation | ThermoFisher (MA5-17123, 31430) |
| MMP9 | Proteolytic regulation | ThermoFisher (MA1-12895, 31430) |
| PDGFB | Pro-fibrotic | ThermoFisher (PA5-19524, 31460) |
| PDGFD | Pro-fibrotic | ThermoFisher (40-2100, 31460) |
| PF4 | Activation indicator | ThermoFisher (MA5-23926, 31430) |
| PPBP | Pro-fibrotic | ThermoFisher (PA5-51254, 31460) |
| TIMP1 | Proteolytic regulation | ThermoFisher (MA1-773, 31430) |
| TIMP2 | Proteolytic regulation | ThermoFisher (MA1-774, 31430) |
| TIMP3 | Proteolytic regulation | ThermoFisher (PA5-26133, 31460) |
| TNFR1 | Activation indicator | ThermoFisher (MA5-23706, 31430) |
| 14-3-3y | Loading control | ThermoFisher (MA1-16587, 31430) |
| Goat Anti-Mouse IgG, HRP | | ThermoFisher (31430) |
| Goat Anti-Rabbit IgG, HRP | | ThermoFisher (31460) |

**Supplemental Table ST6. P-values of independent patient predictors of biomarker measurements by multiple linear regression.**

| Y | Independent Predictors of Y | | | | |
| --- | --- | --- | --- | --- | --- |
|  | BFR | Vintage | Age | Sex | Weight |
| PAF (Plasma) | 0.017 | 0.11 | 0.16 | 0.55 | 0.59 |
| MMP3 (Plasma) | 0.025 | 0.64 | 0.38 | 0.60 | 0.82 |
| MMP14 (Plasma) | 0.12 | 0.038 | 0.31 | 0.25 | 0.88 |
| PAF (ΔPlasma) | 0.018 | 0.066 | 0.83 | 0.15 | 0.10 |
| MMP2 (ΔPlasma) | 0.058 | 0.55 | 0.45 | 0.29 | 0.70 |
| MMP3 (ΔPlasma) | 0.020 | 0.33 | 0.67 | 0.24 | 0.046 |
| TIMP1 (ΔPlasma) | 0.081 | 0.027 | 0.42 | 0.96 | 0.71 |
| IFNg (ΔPlasma) | 0.81 | 0.043 | 0.70 | 0.35 | 0.72 |
| TX11B2 (ΔPlasma) | 0.27 | 0.032 | 0.44 | 0.54 | 0.61 |
| PAFAH2 (ΔRNA) | 0.72 | 0.00019 | 0.16 | 0.83 | 0.37 |
| MMP3 (ΔPL) | 0.046 | 0.63 | 0.089 |  | |
| TIMP2 (ΔPL) | 0.18 | 0.012 | 0.41 |  |  |
| PPBP (ΔPL) | 0.90 | 0.021 | 0.31 |  |  |

Highlighted = p-values < 0.05. Multivariate predictions of ΔPL markers were limited to 3 independent predictors because of sample size. Sex and weight were not found to significantly predict ΔPL measurements in any permutation of ≤ 3 predictors.

**Supplemental Table ST7. Multiple linear regression of plasma clotting factors to MMP and TIMP upregulation in platelets.**

| Predictor(s) | R² | Adjusted R² | P-value |
| --- | --- | --- | --- |
| A = PAF (Plasma), B = 11TXB2 (Plasma), C = PAF (ΔPlasma), D = 11TXB2 (ΔPlasma) | | | |
| y = MMP1 (ΔRNA). y = -0.93 + 2.0E-4*A + 7.6E-5*B | | | |
| A+B | 0.91 | 0.85 | 0.027 |
| A | 0.76 | 0.73 | 0.00050 |
| B | 0.82 | 0.78 | 0.013 |
| y = TIMP2 (ΔRNA). y = -0.93 + 3.3E-5*A + 7.2E-4*B - 1.0*C - 2.5*D | | | |
| A+B+C+D | 0.98 | 0.93 | 0.044 |
| A+B+C | 0.94 | 0.89 | 0.022 |
| A+B+D | 0.97 | 0.95 | 0.0076 |
| A+C+D | 0.58 | 0.46 | 0.028 |
| B+C+D | 0.98 | 0.95 | 0.0061 |
| A+B | 0.94 | 0.92 | 0.0032 |
| A+C | 0.45 | 0.35 | 0.039 |
| A+D | 0.44 | 0.34 | 0.040 |
| B+C | 0.94 | 0.91 | 0.0038 |
| B+D | 0.97 | 0.96 | 0.00080 |
| C+D | 0.11 | 0.023 | 0.31 |
| A | 0.28 | 0.23 | 0.049 |
| B | 0.94 | 0.92 | 0.0004 |
| C | 0.07 | 0.025 | 0.22 |
| D | 0.093 | 0.049 | 0.16 |

**Supplemental Table ST8. Multiple linear regression of platelet upregulation to acute inflammation.**

| Predictor(s) | R² | Adjusted R² | P-value |
| --- | --- | --- | --- |
| A = PDGFD (ΔRNA), B = TBXAS1 (ΔRNA), C = TIMP2 (ΔRNA) | | | |
| y = CRP (ΔPlasma). y = -0.15 - 0.012*A + 0.14*B + 0.34*C | | | |
| A+B+C | 0.87 | 0.82 | 0.00070 |
| A+B | 0.40 | 0.30 | 0.048 |
| A+C | 0.86 | 0.83 | 0.00010 |
| B+C | 0.55 | 0.50 | 0.00040 |
| A | 0.32 | 0.27 | 0.028 |
| B | 0.11 | 0.09 | 0.035 |
| C | 0.49 | 0.47 | 0.0002 |

**Supplemental Table ST9. Multiple linear regression of platelet upregulation to plasma PGI2 levels.**

| Predictor(s) | R² | Adjusted R² | P-value |
| --- | --- | --- | --- |
| A = EGF(ΔRNA), B = GAPDH (ΔRNA) | | | |
| y = PGI2 (Plasma). y = 1.8 + 0.90*A + 0.79*B | | | |
| A+B | 0.82 | 0.70 | 0.078 |
| A | 0.77 | 0.71 | 0.022 |
| B | 0.79 | 0.74 | 0.018 |
| A = PDGFD (ΔRNA), B = TBXAS1 (ΔRNA), C = TIMP2 (ΔRNA) | | | |
| y = PGI2 (ΔPlasma). y = -0.14 + 0.29*A + 0.16*B + 0.07*C | | | |
| A+B+C | 0.28 | 0.21 | 0.015 |
| A+B | 0.24 | 0.20 | 0.0088 |
| A+C | 0.27 | 0.23 | 0.0053 |
| B+C | 0.24 | 0.19 | 0.012 |
| A | 0.24 | 0.22 | 0.0021 |
| B | 0.19 | 0.17 | 0.0073 |
| C | 0.18 | 0.16 | 0.0094 |

**Supplemental Table ST10. Multiple linear regression of fibrotic upregulation to platelet MMP levels.**

| Predictor(s) | R² | Adjusted R² | P-value |
| --- | --- | --- | --- |
| A = TGFb1 (ΔRNA), B = TIMP1 (ΔRNA), C = PPBP (ΔRNA) | | | |
| y = MMP1 (ΔPL). y = -0.45 - 0.81*A + 0.64*B | | | |
| A+B | 0.88 | 0.79 | 0.043 |
| A | 0.68 | 0.61 | 0.042 |
| B | 0.78 | 0.72 | 0.020 |
| y = MMP2 (ΔPL). y = 0.75 + 1.0*A + 1.7*B - 0.29*C | | | |
| A+B+C | 0.92 | 0.88 | 0.0033 |
| A+B | 0.92 | 0.90 | 0.00050 |
| A+C | 0.88 | 0.85 | 0.0015 |
| B+C | 0.92 | 0.89 | 0.00050 |
| A | 0.88 | 0.87 | 0.00020 |
| B | 0.92 | 0.91 | 0.000043 |
| C | 0.80 | 0.78 | 0.0011 |

**Supplemental Table ST11. Multiple linear regression of fibrotic upregulation to intra-dialysis plasma TIMP changes.**

| Predictor(s) | R² | Adjusted R² | P-value |
| --- | --- | --- | --- |
| A = PPBP, B = TGFb1, C = GAPDH, D = TBXAS1, E = TIMP1 (ΔRNA) | | | |
| y = TIMP3 (ΔPlasma). y = 0.13 + 0.21*A + 0.038*B - 0.091*C + 0.36*D + 0.20*E | | | |
| A+B+C+D+E | 0.45 | 0.32 | 0.025 |
| A+B+C+D | 0.43 | 0.32 | 0.016 |
| A+B+C+E | 0.40 | 0.29 | 0.020 |
| A+B+D+E | 0.44 | 0.33 | 0.013 |
| A+C+D+E | 0.45 | 0.35 | 0.010 |
| B+C+D+E | 0.41 | 0.30 | 0.021 |
| A+B+C | 0.31 | 0.22 | 0.033 |
| A+B+D | 0.41 | 0.33 | 0.0075 |
| A+B+E | 0.28 | 0.20 | 0.042 |
| A+C+D | 0.43 | 0.35 | 0.0058 |
| A+C+E | 0.39 | 0.31 | 0.0084 |
| A+D+E | 0.44 | 0.36 | 0.0048 |
| B+C+D | 0.33 | 0.24 | 0.028 |
| B+C+E | 0.37 | 0.29 | 0.013 |
| B+D+E | 0.41 | 0.32 | 0.0086 |
| C+D+E | 0.39 | 0.31 | 0.0093 |
| A+B | 0.27 | 0.22 | 0.020 |
| A+C | 0.3 | 0.24 | 0.013 |
| A+D | 0.41 | 0.36 | 0.0023 |
| A+E | 0.28 | 0.23 | 0.015 |
| B+C | 0.21 | 0.15 | 0.055 |
| B+D | 0.33 | 0.27 | 0.0098 |
| B+E | 0.21 | 0.15 | 0.051 |
| C+D | 0.29 | 0.23 | 0.017 |
| C+E | 0.35 | 0.3 | 0.0047 |
| D+E | 0.39 | 0.33 | 0.0029 |
| A | 0.27 | 0.24 | 0.0046 |
| B | 0.16 | 0.13 | 0.032 |
| C | 0.14 | 0.11 | 0.049 |
| D | 0.27 | 0.24 | 0.0055 |
| E | 0.18 | 0.15 | 0.023 |

**Supplemental Table ST12. Multiple linear regression of plasma MMP changes to platelet PAF upregulation.**

| Predictor(s) | R² | Adjusted R² | P-value |
| --- | --- | --- | --- |
| A = MMP2 (ΔPlasma), B = MMP14 (ΔPlasma) | | | |
| y = PAFAH2 (ΔRNA). y = 0.14 + 1.4*A + 0.66*B | | | |
| A+B | 0.60 | 0.50 | 0.026 |
| A | 0.57 | 0.53 | 0.0070 |
| B | 0.44 | 0.38 | 0.026 |

**Supplemental Table ST13. Multiple linear regression of platelet MMPs and TIMPs to plasma TIMP elevation.**

| Predictor(s) | R² | Adjusted R² | P-value |
| --- | --- | --- | --- |
| A = MMP2 (ΔPL), B = MMP3 (ΔPL), C = TIMP2 (ΔPL) | | | |
| y = TIMP1 (Plasma). y = 1.4E5 + 3.8E4*A + 2.3E4*C | | | |
| A+C | 0.79 | 0.69 | 0.043 |
| A | 0.80 | 0.77 | 0.0011 |
| C | 0.63 | 0.57 | 0.011 |
| y = TIMP2 (ΔPlasma). y = -0.021 - 0.22*B - 0.15*C | | | |
| B+C | 0.70 | 0.58 | 0.050 |
| B | 0.63 | 0.59 | 0.0059 |
| C | 0.34 | 0.25 | 0.098 |

**Supplemental Table ST14. Multiple linear regressions of plasma growth factors to platelet fibrotic production.**

| Predictor(s) | R² | Adjusted R² | P-value |
| --- | --- | --- | --- |
| A = TGFβ1 (ΔRNA), B = CTGF (ΔPL), C = TIMP1 (ΔPL) | | | |
| y = FGF2 (Plasma). y = -1387 + 774*A - 4981*B - 84*C | | | |
| A+B+C | 1.0 | 1.0 | 0.0021 |
| A+B | 0.94 | 0.88 | 0.059 |
| A+C | 0.80 | 0.60 | 0.200 |
| B+C | 0.89 | 0.78 | 0.11 |
| A | 0.18 | 0.14 | 0.030 |
| B | 0.86 | 0.81 | 0.023 |
| C | 0.78 | 0.70 | 0.048 |
| A = IGF1 (Plasma), B = FGF2(ΔPlasma), C = IGF1 (ΔPlasma) | | | |
| y = TIMP1 (ΔPL). y = 2.2 - 3.8E-5*A - 3.3*B + 3.2*C | | | |
| A+B+C | 1 | 1 | 0.036 |
| A+B | 0.95 | 0.89 | 0.053 |
| A+C | 0.96 | 0.93 | 0.036 |
| B+C | 0.91 | 0.87 | 0.0075 |
| A | 0.82 | 0.76 | 0.035 |
| B | 0.91 | 0.89 | 0.001 |
| C | 0.8 | 0.78 | 0.0001 |

**Supplemental Table ST15. Multiple linear regression of plasma fibrotic factors to platelet growth factor upregulation.**

| Predictor(s) | R² | Adjusted R² | P-value |
| --- | --- | --- | --- |
| A = MMP9 (Plasma), B = PDGFD (ΔPlasma), C = PPBP (ΔPlasma) | | | |
| y = FGF2 (ΔRNA). y = -0.37 + 9.9E-5*A + 0.92*B +1.1*C | | | |
| A+B+C | 0.76 | 0.65 | 0.015 |
| A+B | 0.43 | 0.32 | 0.047 |
| A+C | 0.57 | 0.47 | 0.023 |
| B+C | 0.69 | 0.61 | 0.0092 |
| A | 0.33 | 0.28 | 0.026 |
| B | 0.3 | 0.24 | 0.042 |
| C | 0.36 | 0.29 | 0.041 |

**SF1. Biomarkers exhibiting comparable dialysis-to-control plasma ranges.**


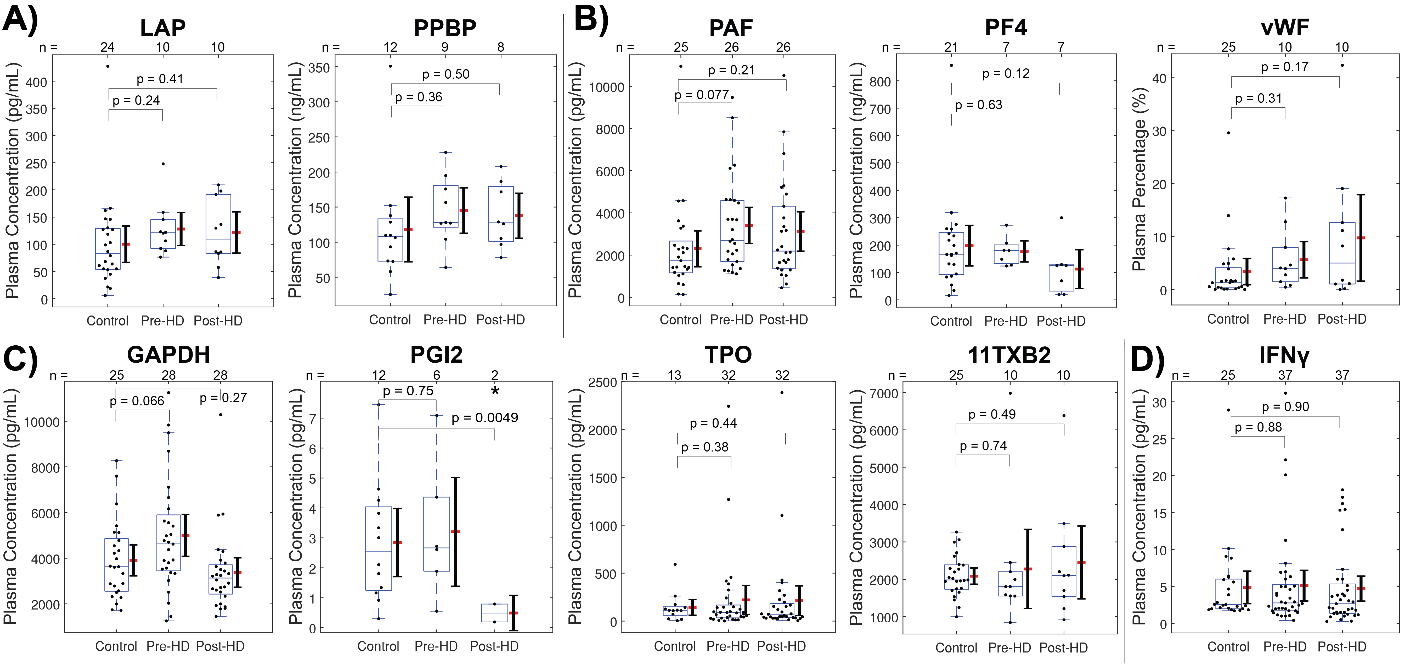


Comparison of plasma concentrations of (**A**) growth factors, (**B**) clotting factors, (**C**) cardiovascular indicators, and (**D**) inflammatory cytokines between healthy control and pre-dialysis or post-dialysis samples as measured by ELISA and compared by t test. *p < 0.05. Box and whisker plots represent quartiles. Adjacent ranges represent mean (red) ± 95% confidence interval. (**C**) Post-dialysis PGI_2_ levels were significantly depressed compared to healthy controls.

**SF2. Plasma biomarker levels unchanged by single dialysis sessions.**


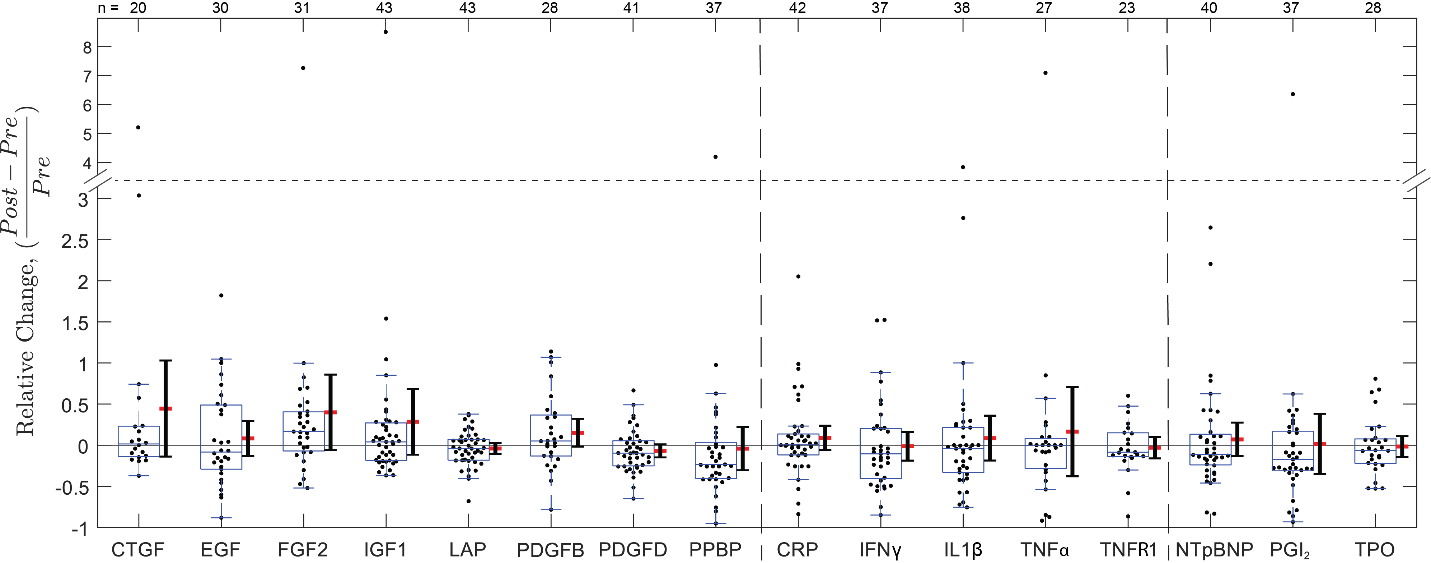


Relative changes in paired plasma samples from dialysis patients pre- and post-treatment as measured by ELISA. Statistical significance was assessed using one-sample t tests. *p < 0.05. Box and whisker plots represent quartiles. Adjacent ranges represent mean (red) ± 95% confidence interval.

**SF3. Multiple linear regression of blood flow rate and patient weight to intra-dialysis changes of plasma MMP3.** **
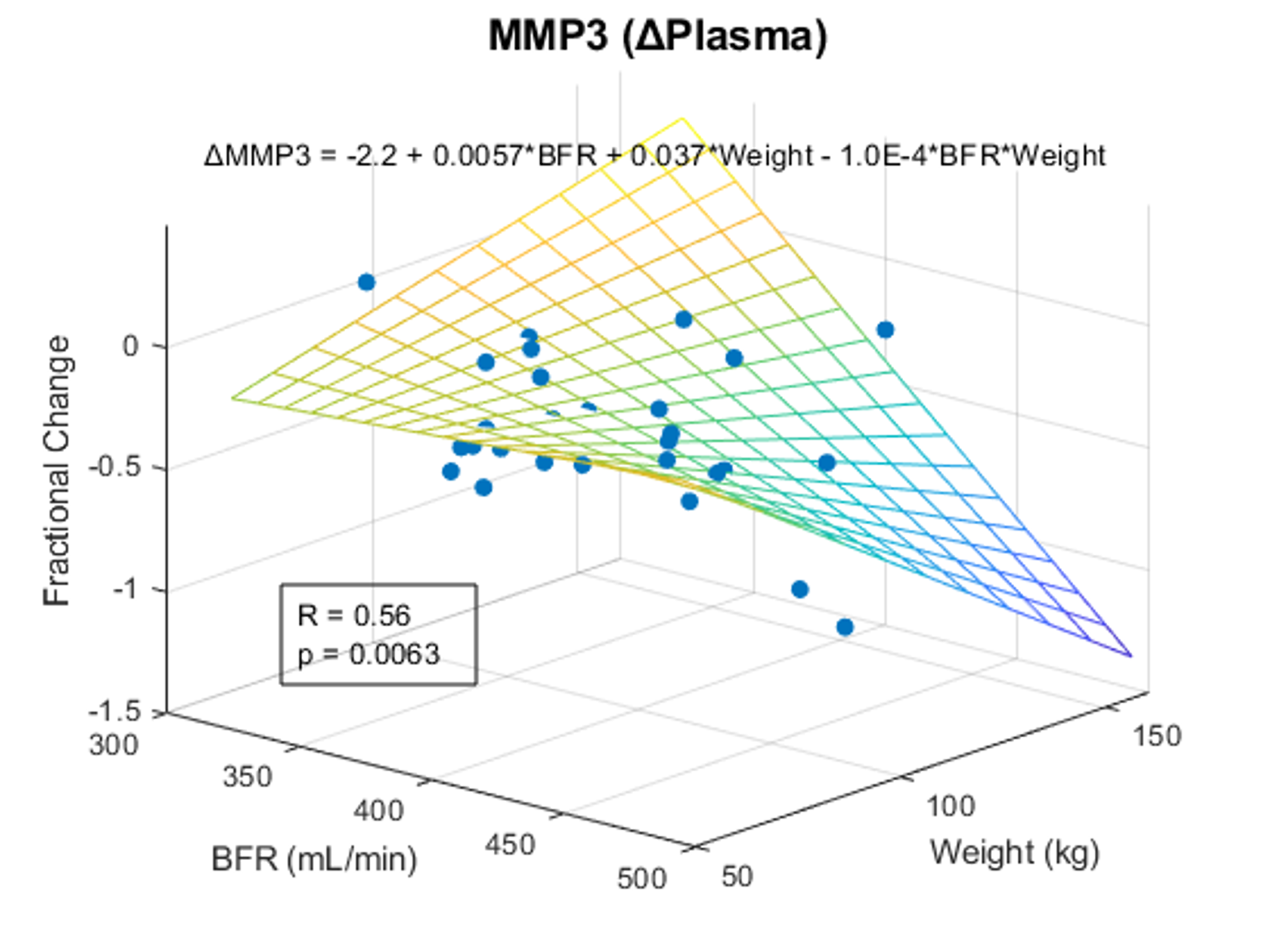
**

Significant multivariate correlation of plasma ΔMMP3 to blood flow rate and patient weight, subtracting interactions between predictors.

**SF4. Raw data to Figure 4 significant correlations.**


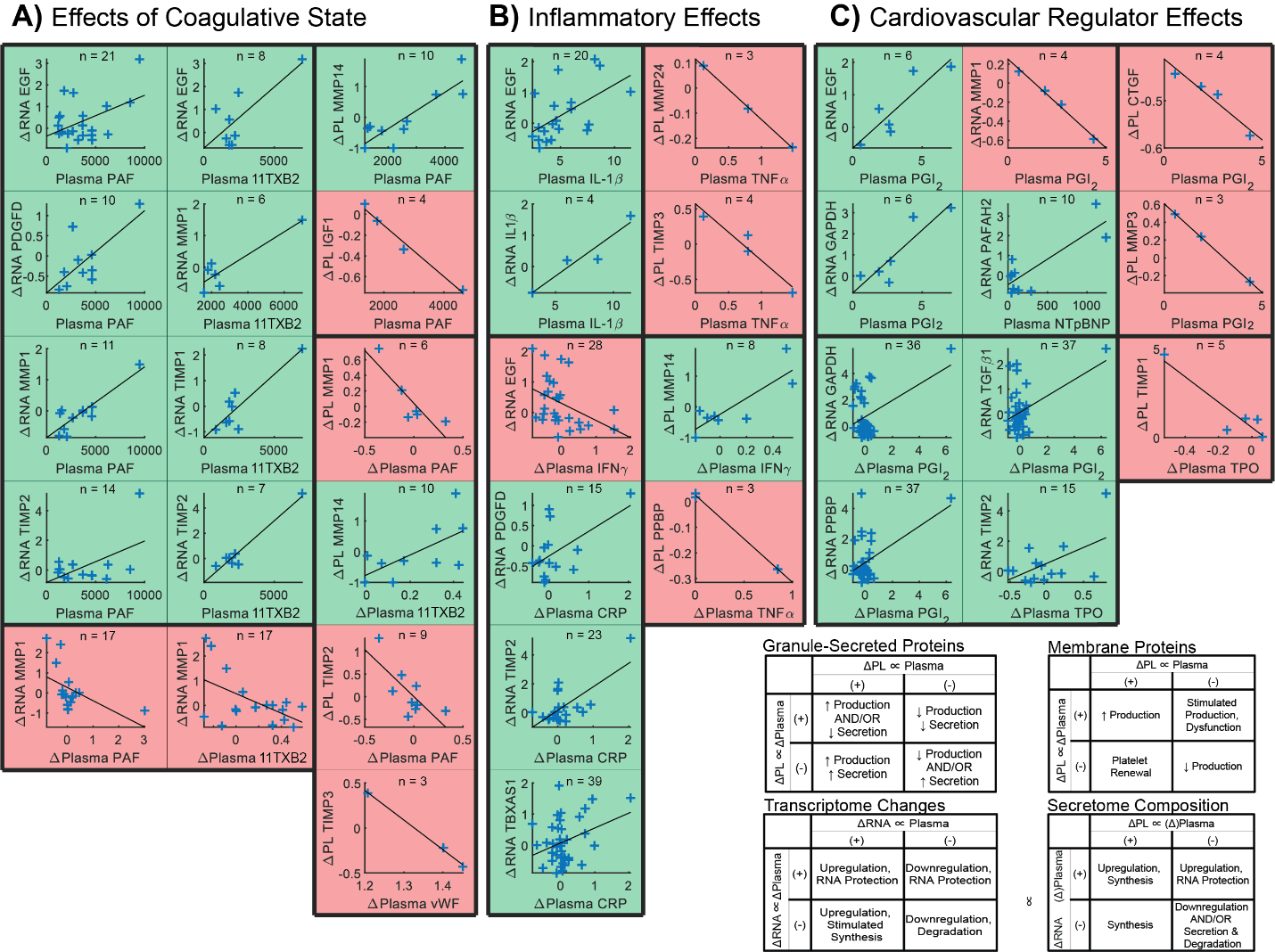


Significant Pearson correlations (p < 0.05) of intra-dialysis platelet lysate RNA or protein relative changes against pre-dialysis plasma protein levels or intra-dialysis plasma relative changes, categorized by interpreted effects on platelets caused by (**A**) coagulative, (**B**) inflammatory, and (**C**) cardiovascular indicators.

**SF5. Raw data to Figure 5 significant correlations.**

Significant Pearson correlations (p < 0.05) of platelet ΔRNA against platelet Δprotein, platelet ΔRNA against Δplasma, and platelet Δprotein against plasma or Δplasma.


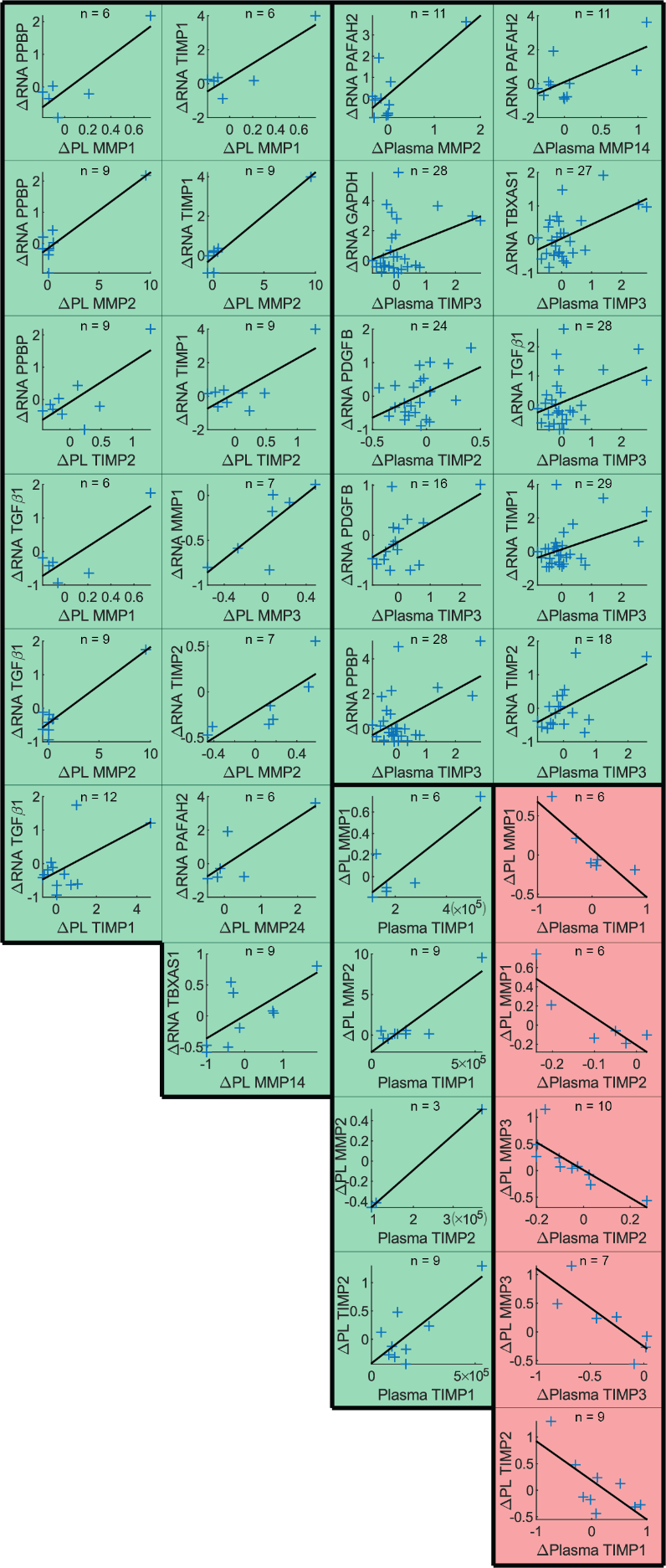


**SF6. Raw data to Figure 6 significant correlations**


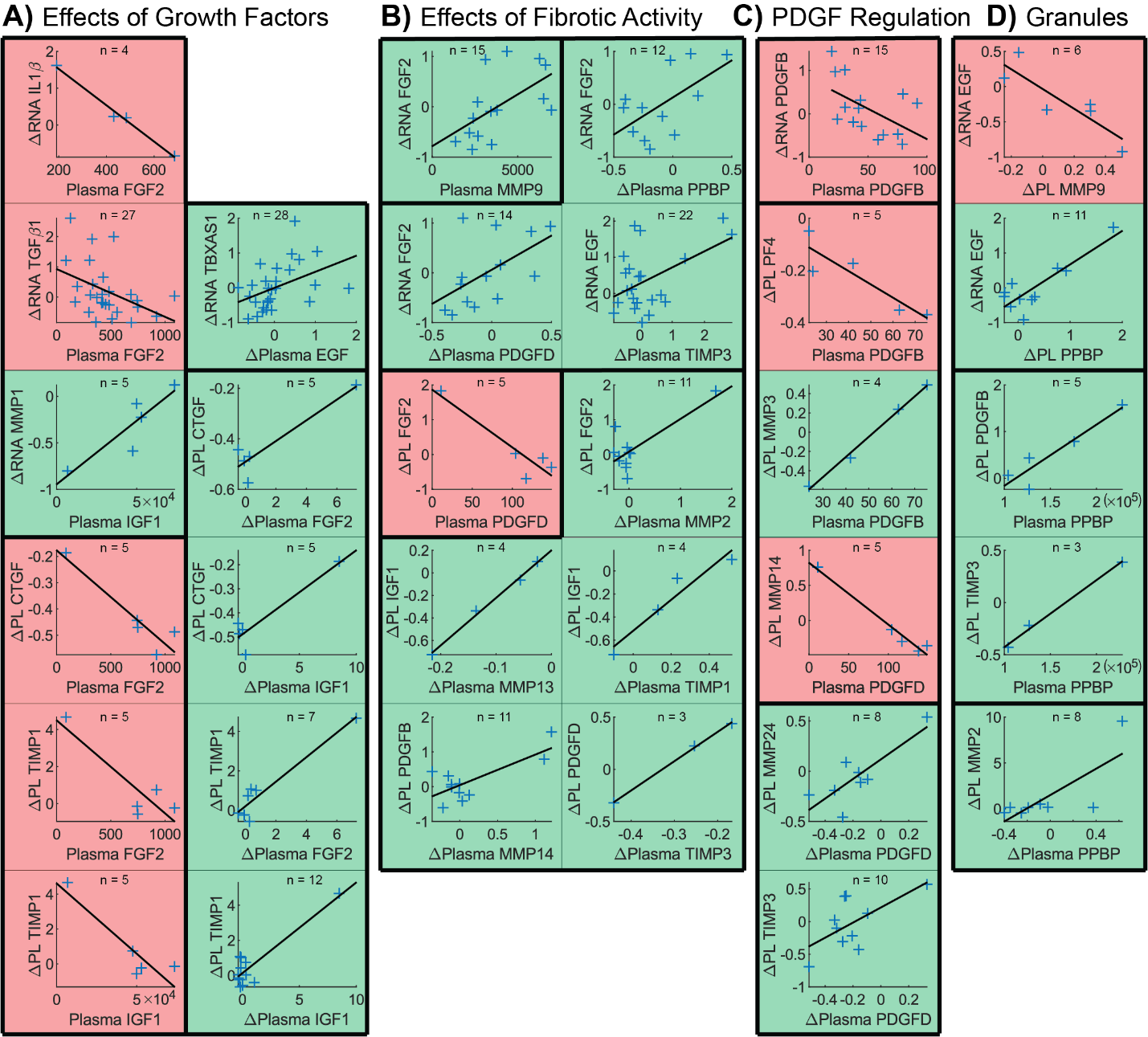


Significant Pearson correlations (p < 0.05) of intra-dialysis platelet lysate RNA or protein relative changes against pre-dialysis plasma protein levels or intra-dialysis plasma relative changes, categorized by interpreted effects on platelets caused by (**A**) growth factors, (**B**) fibrotic activity, and (**C**) PDGFs. (**D**) Correlations amongst alpha-granule and lysosome proteins elucidate physiological adaptations contributing to pro-fibrotic or fibro-protective functions.
